# Myokine-mediated mechanisms of immune checkpoint inhibitors-associated colorectal injury and repair in rectal cancer

**DOI:** 10.3389/fimmu.2026.1852152

**Published:** 2026-08-18

**Authors:** Shuang Ma, Jinxue Rong, Xinwei Wu, Xiaolin Wu, Yibo Gao, Huixian Li, Tao Yan

**Affiliations:** 1 School of Information Science and Engineering, Shenyang Ligong University, Shenyang, China; 2 School of Mathematics and Statistics, Liaoning University, Shenyang, China; 3 Department of Oral and Maxillofacial Surgery, Taikang Bybo Dental, Beijing, China; 4 Department of Anesthesiology, National Cancer Center/National Clinical Research Center for Cancer/Cancer Hospital, Chinese Academy of Medical Sciences and Peking Union Medical College, Beijing, China

**Keywords:** immune checkpoint inhibitors, myokines, rectal cancer, skeletal muscle, tumor microenvironment

## Abstract

Rectal cancer exhibits prominent anatomical and molecular heterogeneity, shaping a tumor microenvironment that determines responsiveness to immune checkpoint inhibitors (ICIs). Though ICIs exert robust anti-tumor effects on dMMR/MSI-H rectal tumors, overactivated immunity causes ICI-associated colorectal injury (ICI-CRI) characterized by diarrhea, mucosal ulceration and even perforation. Its pathological mechanisms cover CD8^+^ T cell-mediated epithelial cytotoxicity, inflammatory cytokine cascades, gut dysbiosis-induced endotoxin translocation, microvascular hypoperfusion, lactic acid accumulation and extracellular matrix fibrosis. Skeletal muscle-secreted myokines (IL-6, IL-15, irisin) form a critical gut-muscle regulatory axis: IL-15 and irisin protect intestinal mucosa by restraining immunosuppressive cells, while excess muscle-derived IL-6 amplifies intestinal inflammation. Multimodal imaging combined with radiomics and artificial intelligence realizes quantitative monitoring of mucosal, vascular and fibrotic lesions. Stratified management schemes including stepwise immunosuppression, microbiota reconstruction, metabolic support and muscle-targeted exercise/nutrition can interrupt the vicious loop linking sarcopenia and intestinal damage. This review systematically illustrates the myokine-centered regulatory network, providing theoretical references to maximize ICI efficacy while reducing gastrointestinal toxicity for personalized rectal cancer immunotherapy.

## 1 Introduction

Colorectal cancer (CRC) ranks among the most prevalent and lethal malignancies worldwide. Its tumorigenesis progresses through multistep pathological cascades driven by the interplay of genetic aberrations, epigenetic modifications, chronic inflammatory activation, and intestinal flora dysbiosis (1). As the predominant subtype of CRC, rectal cancer possesses distinct anatomical features and unique immune microenvironmental profiles that differentiate it from colonic cancer. Although conventional therapeutic strategies, including surgical resection, radiotherapy, and chemotherapy, have improved clinical outcomes for certain patients, these regimens yield limited efficacy in advanced and recurrent cases. Benefiting from advancing insights into tumor immune biology and the widespread clinical application of immune checkpoint inhibitors (ICIs), immunotherapy has emerged as a transformative therapeutic breakthrough for rectal cancer treatment in recent years. In particular, for patients diagnosed with mismatch repair-deficient/microsatellite instability-high (dMMR/MSI-H) colorectal cancer, ICIs substantially enhance therapeutic efficacy and long-term survival by reinvigorating endogenous antitumor immune responses, representing a pivotal paradigm shift from conventional cytotoxic therapy to precision immunotherapy (2). Beyond tumor microenvironment (TME) regulation, skeletal muscle status has emerged as a critical non-tumoral factor closely correlated with systemic immune homeostasis. Functional and healthy skeletal muscle optimizes whole-body metabolic profiles and potentiates immune competence, thereby favorably supporting immunotherapeutic efficacy. Accumulating evidence has demonstrated that deteriorated skeletal muscle quality impairs intestinal immune homeostasis, which further elevates the susceptibility to immune-related gastrointestinal adverse events during Immune Checkpoint Inhibitor (ICI) treatment (3). Collectively, targeting skeletal muscle health holds great potential to optimize comprehensive therapeutic outcomes and provides novel insights and actionable strategies for personalized colorectal cancer immunotherapy. The colon and rectum share common intestinal immune and microbiota regulatory mechanisms. However, owing to the unique anatomical characteristics of the rectum, including abundant mesenteric tissues and a dense extracellular matrix microenvironment, rectal lesions exhibit a more pronounced fibrotic phenotype after injury. Therefore, this study comprehensively elaborates the general pathogenic mechanisms applicable to colorectal carcinoma while highlighting the distinct pathological features specific to rectal tissues. The TME of rectal cancer, consisting of three major components—immune cells, tumor cells, and stromal cells—exhibits significant site specificity and molecular heterogeneity and plays a decisive role in the response to immunotherapy (4). The dMMR/MSI-H subtype forms an immunologically active “hot tumor” microenvironment owing to its high tumor mutational burden and abundant neoantigens, conferring sensitivity to ICIs (5). In contrast, the mismatch repair-proficient/microsatellite stable subtype is characterized by immunosuppressive cell infiltration and a dense extracellular matrix, resulting in a “cold tumor” phenotype that is typically refractory to immunotherapy (6). With the widespread application of immunotherapy, intestinal immune checkpoint inhibitor-associated immune-mediated colorectal injury(ICI-CRI) have increasingly become a focus of clinical research. By inhibiting the programmed cell death protein 1/programmed death ligand 1 (PD-1/PD-L1) or cytotoxic T lymphocyte-associated protein 4 (CTLA-4) pathways, ICIs relieve the immunosuppression imposed by tumors on the immune system, thereby enhancing T cell activity and effectively eliminating tumor cells (7). However, overactivated immune responses may also break through tissue immune tolerance, leading to the misrecognition of intestinal epithelial cells as target cells and triggering severe complications, such as diarrhea, mucosal inflammatory lesions, and even colon perforation (8). This colorectal injury caused by immune imbalance reflects the double-edged sword nature of the TME, involving both immune activation and tissue homeostasis maintenance, and poses new challenges in optimizing immunotherapy strategies. Therefore, an in-depth analysis of the mechanisms of immunotherapy-induced TME remodeling and its role in colorectal injury provides a key scientific basis for formulating precise interventions and individualized treatment strategies (9).

This review systematically examines the composition and biological characteristics of the TME in rectal cancer, elaborates on the regulatory mechanisms of immunotherapy in the TME and the immune-related injuries it induces, and focuses on exploring the pathogenesis of colorectal injury after immunotherapy, imaging evaluation methods, and multi-level repair strategies. Based on the regulatory axis covering skeletal muscle, myokines, intestinal immune homeostasis, and ICI-CRI, this study takes myokines as the core regulatory molecule throughout the full text. It systematically clarifies the molecular mechanisms by which key myokines [interleukin-6 (IL-6), interleukin-15 (IL-15), and irisin] modulate intestinal epithelial integrity, immune cell function, TME remodeling, as well as vascular and fibrotic injury. Combined with multimodal imaging biomarkers and stratified repair strategies, an integrated regulatory network of “skeletal muscle–myokine–intestinal injury–repair” is constructed, which remedies the limitations of existing reviews that merely focus on intestinal immune toxicity or muscle wasting in isolation. By integrating multidisciplinary evidence from immunology, molecular pathology, and radiomics, this study aimed to reveal the pathological processes and repair rules of immunotherapy-related injuries in rectal cancer, provide a theoretical basis and practical guidance for clinical precise intervention and safe reinitiation of immunotherapy, and offer directions for the optimization of individualized immunotherapy strategies in the future.

## 2 Composition, characteristics of rectal cancer TME and its dynamic remodeling in immunotherapy

TME is a pivotal regulator of the pathogenesis, progression, and response to immunotherapy in rectal cancer. The dynamic interplay between the TME and ICIs underpins the mechanistic basis for neoadjuvant immunotherapy. This chapter systematically elaborates on the composition and core characteristics of the rectal cancer TME, as well as the mechanisms of immune remodeling induced by ICIs.

### 2.1 Composition and characteristics of rectal cancer TME

The composition of the rectal cancer TME exhibits significant site specificity and molecular subtype heterogeneity, with its cellular makeup and functional characteristics directly influencing tumor progression and response to immunotherapy. The TME primarily consists of three major components: immune, tumor, and stromal cells. These constituents collectively shape the unique pathological microenvironment of rectal cancer through intricate interactions (10).

The skeletal muscle plays a crucial role in the TME. As the primary organ for locomotion in the body, skeletal muscle not only maintains physical function but also influences immune responses through metabolic regulation and the secretion of cytokines such as IL-6 and IL-15 (11). Research has shown that a healthy state of skeletal muscle is closely associated with antitumor immune responses. Specifically, muscle fibers can release myokines that promote the proliferation and activation of T cells and Natural Killer cell (NK) cells, thereby enhancing the body’s immune response against tumors (12). Consequently, the quality and function of skeletal muscle in patients with rectal cancer may directly affect their response to immunotherapy.

Skeletal muscle-derived myokines consist of three distinct subtypes, including protective irisin and IL-15, as well as the pathogenic molecule IL-6. The muscle-secreted myokines IL-15, irisin and IL-6 jointly regulate the balance between anti-tumor immunity and tissue inflammatory homeostasis by modulating the activation of CD8^+^ cytotoxic T cells, regulatory T cells (Tregs) and M2-type tumor-associated macrophages via inflammatory Janus Kinase (JAK)/Signal Transducer and Activator of Transcription (STAT) and nuclear factor κB (NF-κB) signaling cascades (13). Among muscle-derived cytokines (myokines), irisin, IL-6 and IL-15 are three core functional mediators linking skeletal muscle activity, systemic immunity and intestinal inflammatory homeostasis. IL-15 exerts potent anti-tumor immunomodulatory effects primarily through the JAK/STAT5 signaling cascade; this signaling axis drives the proliferation, intratumoral infiltration of CD8^+^ cytotoxic T cells and NK cells, and ultimately strengthens systemic anti-tumor immune surveillance (14). IL-15 limits the suppressive function of intestinal Tregs, restores the anti-tumor activity of CD8^+^ and NK cells, and relieves immunosuppression in colitis-associated colorectal cancer (15). Irisin acts as an immune-regulatory myokine derived from skeletal muscle and targets tumor-associated macrophages. By inhibiting the STAT3 signaling cascade upstream of Colony-Stimulating Factor 1 (CSF-1), it blocks M2-type Tumor-Associated Macrophage (TAM) polarization, reduces the secretion of immunosuppressive Interleukin-10 (IL-10) and Transforming Growth Factor-β (TGF-β), and relieves local immune suppression within the rectal TME (16). Irisin blocks the upstream STAT3 signaling pathway in M2-TAMs, reduces macrophage-secreted TGF-β, attenuates stroma-derived immunosuppressive signals, and ultimately elevates the infiltration of effector immune cells in tumor tissues. IL-6 derived from colonic intraepithelial lymphocytes maintains intestinal epithelial barrier homeostasis under physiological conditions, protecting colon tissue from inflammatory injury (17). Physiological low concentrations of IL-6 maintain intestinal immune tolerance and epithelial barrier integrity, while excessive IL-6 secretion induced by muscle atrophy activates persistent NF-κB signaling, triggers massive release of pro-inflammatory mediators, and exacerbates intestinal mucosal injury after ICI administration. Under muscle atrophy conditions, excessive IL-6 persistently activates the NF-κB signaling pathway in intestinal epithelial cells. The subsequent overproduction of pro-inflammatory mediators aberrantly activates intestinal CD8^+^ T cells, which trigger cross-recognition of self-antigens and ultimately result in intestinal mucosal injury. Meanwhile, IL-6 facilitates the differentiation of M2-TAMs via the JAK/STAT3 pathway, further amplifying the local immunosuppressive microenvironment (18).

Tumor cells serve as the core drivers within the TME, and their molecular characteristics vary considerably depending on the mismatch repair status. As stated in the introduction, mismatch repair-proficient/microsatellite stable tumors are defined as immune-cold tumors, whereas dMMR/MSI-H tumors are characterized as immune-hot tumors. Immune cells are pivotal components of the TME in rectal cancer and display distinct functional polarization. The functional differentiation of immune cells differs substantially between the two subtypes, and skeletal muscle homeostasis positively modulates the cytotoxic function of tumor-infiltrating immune cells. Therefore, the strength and quality of skeletal muscle may influence immunotherapy effectiveness. Within the immune-hot TME of dMMR/MSI-H tumors, follicular helper T cells and B cells in this subtype frequently assemble into tertiary lymphoid structures to robustly amplify tumor-specific adaptive immune responses. Conversely, the TME of the mismatch repair-proficient/microsatellite stable subtype is characterized by abundant infiltration of immunosuppressive cells, including myeloid-derived suppressor cells, M2-polarized TAMs, and Tregs. These cells suppress effector T cell functionality either by secreting immunosuppressive cytokines such as TGF-β and IL-10, or by depleting essential amino acids like arginine and tryptophan (19). Additionally, neutrophils with elevated NOD-Like Receptor Family Pyrin Domain-Containing 3 (NLRP3) expression, activated via the NF-κB pathway, adopt a pro-tumorigenic phenotype that exacerbates the immunosuppression. These multiple inhibitory signals collectively constitute the immunosuppressive microenvironment of mismatch repair-proficient/microsatellite stable subtypes. In this context, skeletal muscle depletion and atrophy may further weaken the body’s immune defenses, leading to poorer treatment responses. Therefore, focusing on the health status of skeletal muscle and implementing nutritional interventions to improve muscle quality may enhance the response of patients with rectal cancer to immunotherapy. Stromal cells provide critical structural support within the TME of rectal cancer, underscoring the anatomical specificity of the rectum. The mesorectal space is rich in extracellular matrix, where cancer-associated fibroblasts (CAFs) secrete components, such as Collagen Type I Alpha 1 Chain, to form a dense fibrotic network. This structure not only influences the growth of tumor cells but may also affect the metabolic state and immune function of the surrounding skeletal muscles. Through the Platelet-Derived Growth Factor Receptor A/B signaling axis, CAFs recruit additional stromal cells, creating a physical barrier that impedes the efficient infiltration of immune cells (20). This fibrotic characteristic is more pronounced in the mismatch repair-proficient/microsatellite stable subtype and is closely associated with a phenotype of high stromal enrichment (21).

### 2.2 Regulatory mechanism of immunotherapy in rectal cancer TME

**Figure 1:**
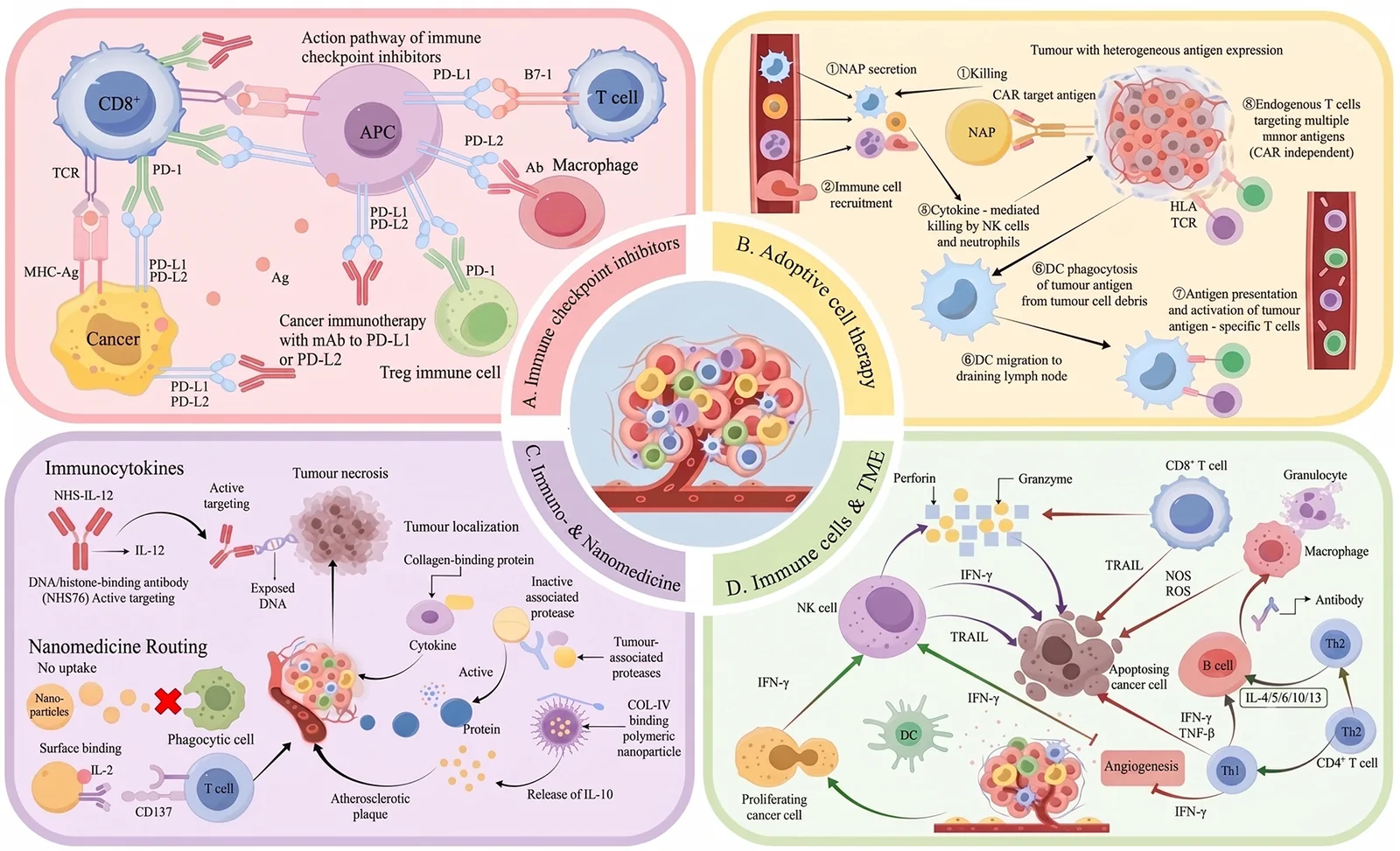
Mechanisms of immunotherapy regulation in the TME. **(A)** ICIs activate immune response; **(B)** Adoptive cell therapy enhances antitumor immunity; **(C)** Cytokines reshape the composition of immune cells; **(D)** Synergistic inhibition between immune cells and the TME.

Inhibition of Immune Checkpoints (PD-1/PD-L1, CTLA-4): ICIs are a class of therapeutic agents that reactivate the body’s antitumor immune response by blocking the interaction between immune checkpoint molecules (such as PD-1/PD-L1 and CTLA-4) and their ligands. Their core mechanism of action is to alleviate the suppression of cytotoxic T cells by the TME, thereby restoring the immune system’s ability to identify and eradicate tumor cells (22). **Figure 1A** schematically illustrates the mechanisms of ICIs: within the interactive network of tumor cells, T cells, and antigen-presenting cells (APCs), the binding of PD-L1 on tumor cells to PD-1 on T cells transmits an inhibitory signal that attenuates T cell-mediated immune attacks. Anti-PD-1/PD-L1 antibodies block this binding, relieving T cell inhibition and promoting the expansion of tumor-infiltrating lymphocytes. Concurrently, the engagement of CTLA-4 on Tregs with Cluster of Differentiation 80/Cluster of Differentiation 86 (CD80/CD86) on APCs also suppresses T-cell activation. CTLA-4 inhibitors disrupt this binding, which not only curbs the immunosuppressive function of Tregs but also enhances co-stimulatory signaling in effector T cells.

Clinical studies have demonstrated that in tumors with high PD-L1 expression, PD-1/PD-L1 inhibitors can abrogate the polarization of M2-type macrophages, reduce the secretion of IL-10, and promote the maturation of APCs (23). Thus, while the PD-1/PD-L1 axis primarily reinvigorates effector T-cells, the CTLA-4 pathway primarily reduces immunosuppressive cell infiltration, creating a dual mechanism of immune disinhibition. Through this coordinated action, ICIs synergistically reshape the immune balance within the TME, ultimately potentiating the body’s antitumor immunity and effectively suppressing tumor growth (19).

In rectal cancer, the spatial architecture of the tumor-stroma interface is critical for determining the response to immunotherapy. Specifically, upon PD-1 blockade, remodeling of the immune cell infiltration pattern at this boundary is essential for enhancing T cell migration toward the tumor core. Research indicates that The spatial organization and immune contexture of the TME are key determinants of the efficacy of immune checkpoint blockade (24).

Relationship Between Skeletal Muscle and Response to Immunotherapy: Skeletal muscle plays a critical role in the TME, particularly by influencing the activity and function of immune cells, which in turn affects the response to immunotherapy. As the primary organ for locomotion, skeletal muscle not only maintains physical function but also regulates immune responses through metabolism and cytokine secretion (25). There is a significant positive correlation between a healthy state of skeletal muscle and antitumor immune responses (26). Specifically, skeletal muscle fibers can release muscle-derived cytokines, known as myokines, such as IL-6 and IL-1 (27). These factors not only promote the proliferation and activation of effector T cells and NK cells but also enhance the antitumor activity of tumor-infiltrating lymphocytes (28). Therefore, skeletal muscle quality is an important factor in regulating immune responses.

In the context of immunotherapy, the status of the skeletal muscle may influence patients’ tolerance and response to treatment. For instance, the strength and function of skeletal muscles in patients with rectal cancer have been found to be closely associated with the efficacy of ICIs (29). Maintaining a healthy skeletal muscle condition can improve systemic inflammatory responses and reduce the formation of an immunosuppressive environment, thereby enhancing the effectiveness of antitumor immunity (30). Conversely, the loss of skeletal muscle, known as sarcopenia, is associated with chemotherapy toxicity and poor prognosis (31). Moreover, combining nutritional interventions targeting muscle loss with appropriate exercise regimens may improve immune cell function and increase their infiltration into the TME. Thus, understanding the role of skeletal muscle in the TME, especially in the context of immunotherapy, is of paramount importance for developing more effective and personalized treatment strategies for cancer. Myokines can further synergistically amplify excessive intestinal immune activation and injury induced by ICI intervention by upregulating PD-L1 expression in tumor and intestinal epithelial cells and modulating Treg cell infiltration.

Penetration and Activation of Adoptive Cell Therapy (CAR-T, TCR-T) in TME: Adoptive Cell Therapy (ACT) represents an innovative modality in cancer immunotherapy. This approach entails isolating, genetically modifying, and expanding a patient’s immune cells (typically T cells) ex vivo, followed by reinfusion to bolster the immune system’s ability to recognize and eradicate tumors (32). **Figure 1B** schematically delineates the processes of tumor infiltration and activation of the two principal adoptive cell therapies, CAR-T and TCR-T, within the TME. CAR-T cells are engineered to degrade Extracellular Matrix (ECM) components, including type I collagen and hyaluronic acid, thereby breaching physical barriers and facilitating their migration to tumor sites (33). This migratory capacity is enhanced by chemokine receptors (e.g., C-C Motif Chemokine Receptor 5) expressed on CAR-T cells, which respond to tumor-derived chemokines (e.g., C-C Motif Chemokine Ligand 5). In contrast, TCR-T cell activation is strictly Major Histocompatibility Complex (MHC)-dependent. Upon recognition of their cognate antigen, TCR-T cells secrete interferon-γ (IFN-γ), which recruits NK cells. Through mechanisms involving the secretion of mediators, such as neutrophil-activating peptides and the recruitment of other immune effectors, TCR-T cells target tumor-associated antigens and activate a broader tumor-specific T-cell response (34). Additionally, the IFN-γ secreted by TCR-T cells can downregulate PD-L1 expression on tumor cells, thereby compromising the critical immune evasion pathways. Thus, TCR-T cells not only mount a direct antitumor attack but also orchestrate innate immune engagement to amplify the overall response (35). Within the TME, CAR-T cells undergo functional potentiation through sustained interactions with tumor cells, leading to their local accumulation and a progressively intensified immune response. Concurrently, activated TCR-T cells promote the trafficking of APCs to lymph nodes, which, in turn, primes and expands antigen-specific T cell populations. However, a significant challenge is that the immunosuppressive solid TME can attenuate TCR-T cell activity. To overcome this, combination regimens with ICIs have been frequently explored to alleviate T cell exhaustion and augment the antitumor potency of TCR-T cells. Collectively, ACT remodels the TME via a multi-mechanistic program encompassing immune cell recruitment, functional activation, and direct tumor cytolysis, thereby establishing a coordinated and synergistic antitumor immune response (21). In the context of rectal cancer, investigative efforts for CAR-T and TCR-T therapies have concentrated on targeting tumor-specific antigens, such as Carcinoembryonic Antigen and Guanylate Cyclase 2C. A paramount research focus involves devising strategies to overcome the impediment of the densely fibrotic mesorectal compartment to enhance T cell penetration (36). Skeletal muscle-derived IL-15 effectively enhances the infiltration efficiency of CAR-T/TCR-T cells in intestinal tissues, relieves the infiltration blockade caused by fibrotic barriers, and facilitates the anti-tumor efficacy of adoptive immunotherapy.

Regulation of Immune Cells by Cytokine Therapy (IL-2, IL-12, IFN-γ): Cytokine therapy is a therapeutic approach that uses cytokines to regulate the immune system, thereby enhancing the body’s immune response against diseases such as tumors. Its main function is to reshape the immune cell composition within the TME by influencing the proliferation, differentiation, activation, and function of immune cells, ultimately strengthening the body’s antitumor immunity (37). As illustrated in **Figure 1C**, cytokines play a crucial role in the TME by forming a complex immunoregulatory network through interactions with immune and tumor cells. Taking IL-12 as example, in adoptive cell therapy, immune cytokines and DNA-nanoconjugated antibodies act synergistically to achieve active targeting, thereby facilitating tumor localization. Collagen-binding proteins in the TME are involved in these mechanisms (38). The figure also illustrates the mechanisms related to tumor necrosis: circulating cytokines bind to nanomolecules, with the unabsorbed fractions being labeled. Nanoparticles interact with phagocytes via their surfaces, and these phagocytes exhibit an alternative phenotype that participates in cytokine production. In this process, inert cytokine precursors are activated by tumor proteases to release the active IL-12. Additionally, nanoparticles conjugated with CD-IV-binding peptides also participate in IL-12 release, thereby enhancing the antitumor immune response (24). This targeted delivery system is particularly suitable for improving the immunosuppressive microenvironment in the mesorectal region of rectal cancer, as it reverses lactate-mediated immunosuppression by activating effector T-cell function. In the TME of rectal cancer, IL-12 and IFN-γ significantly promote the activation of tumor-infiltrating T cells and reverse lactate-mediated immunosuppression. Myokine IL-6 interacts with locally produced tumor necrosis factor-α (TNF-α) and IL-1β in the intestine, forming a continuously amplified inflammatory cascade that further exacerbates intestinal epithelial apoptosis and intestinal immune injury.

Synergistic Inhibition Between Immune Cells (CD8+ T Cells, NK Cells, DCs) and TME: Immune cells play a crucial role in the TME and act synergistically through multiple mechanisms to inhibit tumor growth and progression. **Figure 1D** illustrates the interactions between several common immune cells and the TME. CD8+ T cells precisely recognize antigens on the surface of tumor cells via their T cell receptors (TCRs) and secrete perforin and granzymes to directly kill the tumor cells (39). NK cells release granzymes and perforin and bind to Fas on the surface of tumor cells through Fas Ligand to trigger tumor cell apoptosis. They also activate other immune cells by secreting IFN-γ and TNF-α (40). Macrophages in the TME can polarize into the M1 subtype, which phagocytoses tumor cell debris and secretes pro-inflammatory cytokines. Some TAMs polarize into the M2 subtype, secreting immunosuppressive factors, such as IL-10, to inhibit the immune response (41). After maturation, DCs efficiently present tumor antigens to T cells, activating and guiding T cells to migrate to tumor sites. Additionally, IL-12 secreted by DCs promotes the differentiation of T helper 1 cell (42). Regulatory T (Treg) cells limit the intensity of the immune response through direct contact or by secreting inhibitory cytokines, such as TGF-β and IL-10, thereby maintaining immune tolerance (43). B cells produce antibodies against tumor-specific antigens to label the tumor cells for phagocyte recruitment. Simultaneously, they activate the complement system and enhance the cytotoxic activity of NK cells (44). Meanwhile, some tumor cells are eliminated under the attack of immune cells. Residual tumor cells upregulate immunosuppressive molecules, such as PD-L1, to inhibit T cell activity and release factors, such as TGF-β and IL-10, to further reshape the TME and maintain an immune escape microenvironment. Through their bidirectional regulatory effect of promoting or inhibiting tumor progression, these immune cell subsets constitute the core regulatory unit of TME immune balance and are key targets for immunotherapeutic intervention. The distribution of immune cells in the TME of rectal cancer exhibits spatial heterogeneity: CD8^+^ T cells often accumulate at the tumor-stroma interface, whereas the number of NK cells is comparatively low. Dysregulated myokine expression disrupts the immune homeostasis of CD8^+^ T cells, NK cells and DC cells, thereby further exacerbating ICI-induced intestinal autoimmune injury.

## 3 Colorectal-related injury caused by immunotherapy

### 3.1 Core mechanisms of intestinal injury induced by ICIs

#### 3.1.1 T cell-mediated targeted killing of intestinal epithelium

**Figure 2:**
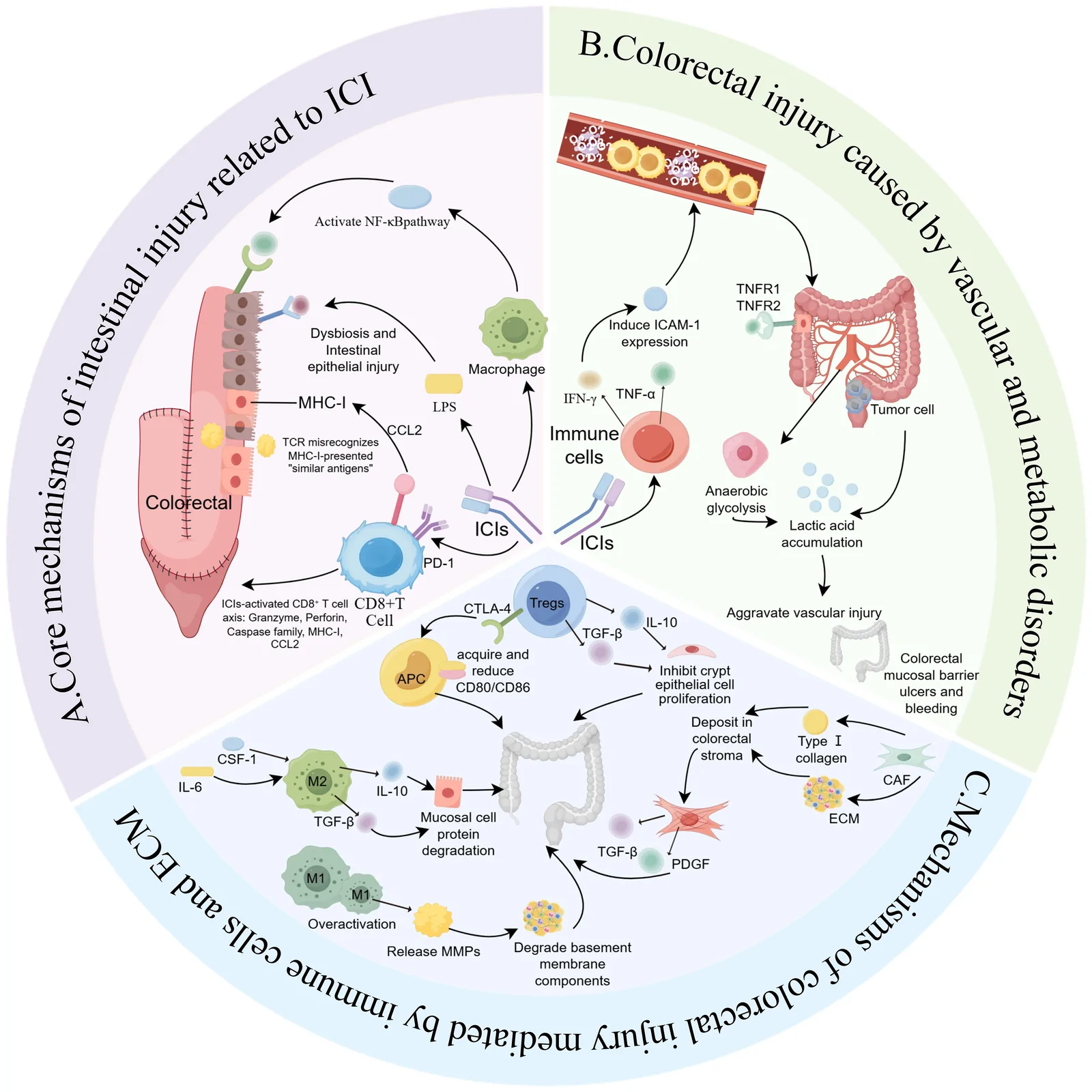
Colorectal injury induced by immunotherapy for rectal cancer. **(A)** Core mechanism of ICI-associated intestinal injury; **(B)** colorectal injury caused by immunotherapy-mediated vascular and metabolic disorders; **(C)** colorectal injury mediated by tregs, M2-type tumor-associated macrophages, and ECM.

The core function of ICIs is to block the PD-1/PD-L1 signaling pathway. Under normal circumstances, the interaction between PD-L1 on tumor cells and PD-1 on T cells inhibits T cell function, prevents their activation, and consequently impairs the antitumor immune response (39). When ICIs bind to PD-1 or PD-L1, this inhibitory pathway is blocked, leading to the reactivation of previously suppressed CD8^+^ T cells (cytotoxic T cells), which regain their capacity to proliferate, migrate, and kill the target cells (**Figure 2A**). For activated CD8^+^ T cells to recognize target cells, they rely on the precise interaction between TCRs and major histocompatibility complex class I (MHC-I) molecules. When the TCR of CD8^+^ T cells binds to antigens similar to those presented by MHC-I on intestinal epithelial cells, the CD8^+^ T cells mistakenly identify the intestinal epithelial cells as abnormal targets for clearance. Subsequently, guided by the chemokines C-C Motif Chemokine Ligand 2 and C-X-C Motif Chemokine Ligand 10, they migrate directionally to the lamina propria of the intestinal mucosa (**Table 1**). After reaching the target area, CD8^+^ T cells release two core cytotoxic molecules: perforin forms pores approximately 1–2 nm in diameter on the membrane of intestinal epithelial cells, disrupting membrane integrity, causing leakage of intracellular contents such as ions and proteins, and triggering cellular osmotic imbalance; granzyme enters the interior of epithelial cells through these perforin-formed pores, activates caspase family proteases, and initiates the cascade reaction of apoptosis (58). Consequently, intestinal epithelial cells undergo lysis due to apoptotic program activation, resulting in local mucosal damage. The activation of cytotoxic CD8^+^ T lymphocytes and their secretion of cytotoxic effector molecules contribute substantially to intestinal epithelial damage and local mucosal injury during ICI-related intestinal toxicity (59). Aberrant IL-6 secretion activates the JAK1–Signal Transducer and Activator of Transcription 3 (STAT3) axis, drives intestinal mucosal accumulation and pro-inflammatory differentiation of CD8^+^ T cells, and exacerbates ICI-related colonic mucosal injury (60). This pathological cascade continuously exacerbates intestinal immune toxicity, ultimately forming a vicious feedback loop between progressive muscle wasting and aggravated intestinal immune damage.

**Table 1:** Core mechanisms of immunotherapy-related injuries in rectal cancer.

Primary injury classification	Secondary injury pathway	Core effector cells & Key molecules	Core signaling pathways	Target sites	Characteristic pathological lesions	References
T cell-mediated intestinal epithelial injury	Cross-recognition and cytotoxic killing of autoantigens	CD8^+^ T cells, perforin, granzymes, CCL2, MHC-I	PD-1/PD-L1 blockade pathway, TCR-MHC recognition pathway	Lamina propria of intestinal mucosa, intestinal epithelial cells	Apoptosis and lysis of epithelial cells, focal mucosal erosion	(45–48)
Inflammatory cytokine storm and intestinal barrier collapse	TNF-α-induced tight junction disruption	Macrophages, neutrophils, TNF-α, occludin, claudin-1	NF-κB signaling pathway	Intercellular tight junctions of epithelial cells	Dissolution of tight junctions, elevated intestinal permeability	(45, 47, 48)
	IL-1β-mediated crypt epithelial apoptosis	NLRP3 inflammasome, IL-1β, caspase family	NLRP3 inflammasome activation pathway	Intestinal crypt epithelial cells	Defective crypt structure, crypt abscess, mucosal rupture	(47–49)
Intestinal flora dysbiosis	Secondary inflammatory amplification induced by LPS translocation	Probiotics/pathogenic bacteria, LPS, TLR4	TLR4-NF-κB cascade pathway	Full-thickness intestinal mucosa	Reduced abundance of beneficial bacteria, LPS translocation, sustained inflammatory amplification	(47, 48, 50)
Microvascular injury and metabolic disorders	Endothelial damage and microthrombosis formation	IFN-γ, TNF-α, ICAM-1, platelets, TNFR1/2	NF-κB, MAPK pathways	Colorectal microvascular endothelium	Luminal stenosis, widened endothelial gaps, microthrombosis, tissue hypoxia	(45, 51)
	Metabolic dysfunction caused by lactic acid accumulation	Lactic acid, monocarboxylate transporters, mitochondria	Anaerobic glycolysis, mitochondrial suppression pathway	Intestinal epithelial cells, crypt stem cells	Tissue acidification, insufficient ATP synthesis, impaired epithelial repair	(45, 51)
Immune suppressor cell and ECM fibrotic injury	Treg-mediated repair suppression	Tregs, CTLA-4, TGF-β, IL-10	CTLA-4 co-stimulatory inhibitory pathway, TGF-β/Smad pathway	Antigen-presenting cells, crypt progenitor cells	Suppressed effector T cell activation, arrested epithelial regeneration	(47, 48, 50)
	Sustained injury amplification by M2-TAMs	M2-TAMs, IL-6, CSF-1, IL-10	JAK/STAT3 pathway	Intestinal epithelial cells, intestinal progenitor cells	Degradation of epithelial structural proteins, autoimmune cross-attack	(45, 51)
	Abnormal ECM deposition and fibrosis	CAFs, type I collagen, fibronectin, MMPs	Integrin α5β1, TGF-β/PDGF pathways	Extracellular matrix, intestinal stem cell niche	Interstitial fibrosis, disrupted stem cell adhesion niche, irreversible scarring	(49, 52, 53)
ICI-induced skeletal muscle injury	Immune cell-triggered myocyte apoptosis	CD8^+^ T cells, NK cells, TNF-α, IFN-γ	NF-κB, JAK/STAT pathways	Skeletal muscle cells, tendon basement membrane	Myocyte apoptosis, protein degradation, impaired muscle repair, sarcopenia	(54–57)

#### 3.1.2 Inflammatory cytokine storm and intestinal barrier collapse

Activated macrophages and neutrophils accumulate extensively in the intestinal mucosa and release pro-inflammatory factors such as tumor necrosis factor-α (TNF-α), IL-6, and IL-1β. These factors collectively damage the intestinal barrier through distinct pathways, ultimately leading to its collapse and mucosal injury (**Table 1**). As shown in **Figure 2A**, after TNF-α binds to receptors on intestinal epithelial cells, it activates the intracellular nuclear factor κB (NF-κB) signaling pathway. The activated NF-κB pathway inhibits the expression of the tight junction proteins occludin and claudin-1 in the intestinal epithelium. Reduced expression of occludin and claudin-1 leads to widened gaps between adjacent epithelial cells, diminishing the barrier’s ability to block substances, significantly increasing permeability, and compromising the structural integrity of the intestinal barrier (61). After IL-1β binds to receptors on intestinal epithelial cells, it activates the intracellular NLRP3 inflammasome. Activation of the NLRP3 inflammasome initiates the cellular apoptosis program, thereby impairing the normal survival and function of intestinal crypt epithelial cells (45). Inflammatory factor storm-mediated intestinal barrier disruption represents another core local pathogenic pathway of ICI-induced intestinal toxicity. Aberrant myokine secretion amplifies intestinal damage via exacerbating inflammatory storm and intestinal barrier dysfunction, thereby establishing a vicious cycle between muscle wasting and progressive intestinal immune toxicity.

#### 3.1.3 Synergistic aggravation of intestinal dysbiosis

ICIs can disrupt the immune tolerance equilibrium between the host and intestinal flora. Under physiological conditions, the host immune system does not mount an immune attack against commensal intestinal bacteria, thereby maintaining a stable microbial community. However, after ICI treatment relieves immunosuppression, an overactivated immune system may erroneously generate an immune response against beneficial bacteria, inhibiting their survival and proliferation (44). Following the disruption of this balance, the levels of beneficial bacteria, including Faecalibacterium prausnitzii, decrease markedly, whereas pathogenic bacteria, such as Enterococcus faecalis, proliferate excessively owing to reduced competition, resulting in a profound imbalance in the composition and proportion of intestinal flora (62). Among the depleted protective commensal bacteria, Akkermansia muciniphila and Bifidobacterium serve as two pivotal beneficial intestinal symbionts. Beneficial commensal bacteria such as Akkerman Sia municipia can reshape and repair the intestinal mucosal mucus barrier by boosting goblet cell differentiation (63). Gut symbionts participate in the systemic regulation of skeletal muscle-derived myokine profiles via the gut–muscle axis, and balanced intestinal microbiota restrains excessive muscle inflammation-associated myokine secretion (64). Such effects block the persistent activation of the intestinal epithelial Toll-like receptor 4 (TLR4)/NF-κB inflammatory cascade, alleviate intestinal mucosal inflammatory infiltration and epithelial damage induced by ICI treatment, and exert definite protective effects against ICI-associated colitis. Akkermansia muciniphila serves as a protective commensal strain that reinforces intestinal mucus barrier and alleviates colonic inflammatory injury (63).

Pathogenic bacteria release lipopolysaccharides (LPS) during proliferation. Concurrently, the intestinal barrier, already compromised by prior inflammatory damage with increased permeability, allows LPS to translocate across the intestinal epithelium into the mucosal tissues (**Figure 2A**). Translocated LPS binds to TLR4 on mucosal cells, activating the intracellular NF-κB signaling pathway (40). NF-κB activation triggers the sustained release of pro-inflammatory factors, including IL-6 and TNF-α. Relying on the above mechanisms of gut dysbiosis, endotoxin translocation and NF-κB inflammatory cascade, aberrant release of myokines can further aggravate intestinal mucosal inflammatory injury, thereby forming a vicious cycle of muscle-derived inflammation and gut microbiota disturbance.

### 3.2 Mechanism of colorectal injury caused by immunotherapy-mediated vascular abnormalities and metabolic disorders

Immune cells activated by immunotherapy release pro-inflammatory cytokines, such as TNF-α and Interferon-γ (IFN-γ), which act directly on the colorectal microvascular system and disrupt its normal structure and function (**Table 1**). IFN-γ induces the expression of the adhesion molecule Intercellular Adhesion Molecule-1 on colorectal microvascular endothelial cells, promoting the extensive adhesion of inflammatory cells, such as neutrophils and lymphocytes, to the vascular wall, thereby narrowing the microvascular lumen (65). Simultaneously, TNF-α binds to its specific receptors, Tumor Necrosis Factor Receptor 1 and Tumor Necrosis Factor Receptor 2, on the surface of colorectal microvascular endothelial cells, recruits signaling complexes, and rapidly activates intracellular NF-κB and Mitogen-Activated Protein Kinase (MAPK) pathways (**Figure 2B**). Activation of these signaling pathways directly targets the interendothelial barrier structure. Phosphorylation modifications mediated by NF-κB/MAPK cause conformational changes, ubiquitin-dependent degradation, or endocytosis from the plasma membrane into the cytoplasm of core proteins such as occludin, claudin-5, and Zonula Occludens-1, resulting in the dissolution of tight junctions and widening of intercellular gaps (66). Based on the aforementioned pathways of gut microbiota dysbiosis, endotoxin translocation, and NF-κB inflammatory cascade, aberrant myokine release further aggravates intestinal inflammatory injury, thereby constructing a vicious cycle between myogenic inflammation and intestinal flora disturbance. Furthermore, the combined action of IFN-γ and TNF-α promotes platelet activation and aggregation, forming microthrombi within the microvessels and further obstructing the vascular lumen. This impedes the normal transport of oxygen and nutrients by microvessels in the colorectal mucosa and submucosa, causing colorectal cells to enter a state of hypoxia and nutrient deficiency. Due to inadequate energy supply, mucosal cells gradually experience functional impairment and may even undergo apoptosis, eventually leading to mucosal erosion (67).

The interplay between hypoxia induced by vascular dysfunction and tumor metabolic reprogramming (the Warburg effect) further exacerbates colorectal metabolic disorders (68). Hypoxia compels cells to shift from aerobic metabolism to anaerobic glycolysis, and the resultant massive accumulation of lactic acid lowers tissue pH, damages cell membranes, inhibits metabolic enzyme activity, and compromises the mucosal barrier (**Figure 2B**). Concurrently, tumor cells release lactic acid via monocarboxylate transporters, aggravating local acidification and mitochondrial suppression, which reduces Adenosine Triphosphate production and hinders the proliferation and repair capacity of crypt cells (69). Based on the full pathological pathways of microvascular endothelial injury, microthrombosis formation, and lactic acid accumulation-induced metabolic disorders described above, dysregulated myokine secretion further aggravates hypoxic damage in the colorectal mucosa, thereby triggering a vicious cycle between muscle wasting and intestinal vascular-metabolic dysfunction.

### 3.3 Mechanism of colorectal injury mediated by Tregs, M2-type TAMs, and ECM in immunotherapy

During immunotherapy, Tregs and M2-type Tumor-Associated Macrophage (TAMs) in the colorectal TME synergistically amplify tissue damage through cytokine signaling, whereas the Extracellular Matrix (ECM) interacts with immune cells to inhibit repair processes (**Table 1**). Tregs highly express CTLA-4, which, on one hand, sequesters and downregulates Cluster of Differentiation 80/Cluster of Differentiation 86 (CD80/CD86) molecules on antigen-presenting cells, thereby weakening the activation signals for effector T cells; on the other hand, Tregs directly secrete Transforming Growth Factor-β (TGF-β) and Interleukin-10 (IL-10), which inhibit the proliferation and differentiation of colorectal crypt epithelial cells and impede mucosal repair (70). M2-type TAMs are polarized under the influence of IL-6 and CSF-1. The IL-10 they secrete activates the Janus Kinase (JAK)/STAT3 signaling pathway in intestinal epithelial cells, upregulating ubiquitin ligase expression and accelerating the degradation of structural proteins in mucosal cells (**Figure 2C**). Concurrently, resident M2-type TAMs continuously secrete TGF-β to suppress the differentiation of intestinal progenitor cells, whereas IFN-γ released by effector T cells enhances MHC-I molecule expression on normal intestinal epithelial cells, rendering them more susceptible to misguided T cell attacks and establishing a vicious cycle (71).

Abnormal ECM deposition and immune cell infiltration further suppress colorectal repair processes. Although the colon and rectum share conserved regulatory pathways of intestinal stromal activation and fibrosis with highly consistent core mechanisms in immune microenvironment disturbance, stromal cell activation, and ECM remodeling, the rectum exhibits unique anatomical characteristics with abundant mesenteric tissues and compact stromal structure. These distinct features contribute to markedly higher activation of cancer-associated fibroblasts (CAFs), excessive collagen deposition, and intensified stromal fibrotic remodeling in rectal tissues compared with colonic tissues (72). Based on such pathological differences, myokines specifically target rectal stromal remodeling processes and exert more sensitive and critical regulatory effects on rectal ECM structural disruption and fibrosis progression. Accordingly, myokine signaling serves as a vital upstream mechanism driving rectal-specific fibrotic injury and aggravating the impaired repair of rectal mucosa (73). CAFs secrete type I collagen and fibronectin, which are abnormally deposited in the colorectal stroma. Via the integrin α5β1 (α5β1) signaling pathway, intestinal fibroblasts are activated and transformed into myofibroblasts. These myofibroblasts secrete TGF-β and Platelet-Derived Growth Factor, promoting submucosal fibrosis that physically impedes epithelial regeneration (74) (**Figure 2C**). Immune cells concurrently disrupt the repair microenvironment: CD8^+^ T cells release perforin and granzymes, directly killing intestinal epithelial cells and expanding the area of mucosal damage; overactivated M1-type macrophages release matrix metalloproteinases (MMPs), which degrade ECM constituents, including laminin and collagen IV, thus disrupting the adhesion niche for intestinal stem cells and reducing their proliferative capacity. Based on the comprehensive pathological pathways involving excessive activation of immunosuppressive cells, aberrant extracellular matrix deposition, and fibrosis-mediated repair arrest, excessive myokine secretion further impedes the repair process of intestinal mucosa, ultimately establishing a vicious cycle of myogenic inflammation and intestinal fibrotic injury.

### 3.4 Mechanisms of immunotherapy-induced damage to skeletal muscle

Immunotherapy not only affects the intestinal system but may also cause damage to the skeletal muscle. This is primarily achieved through inflammation elicited by immune activation and direct attack on skeletal muscle cells (75). Activated CD8^+^ T cells and associated inflammatory cells can target skeletal muscle tissue that expresses specific cytokines or antigens, leading to the programmed cell death of muscle cells. Additionally, immune cells, such as NK cells, may contribute to skeletal muscle damage (76). Consistent with the intestinal fibrotic injury pathways mediated by Tregs, M2-TAMs, and ECM as illustrated in Chapter 3.3, dysregulated myokine expression synchronously exacerbates the pathological progression of intestinal fibrosis and triggers autoimmune injury in skeletal muscle. Similar to its effects on the intestine, inflammation-mediated damage to the skeletal muscle can lead to secondary systemic muscle disorders and metabolic issues. For example, the role of skeletal muscle in energy metabolism may be diminished, affecting the metabolism of glucose and fats, which can ultimately impact overall health and lead to fatigue and functional decline (77). Integrating the aforementioned intestinal injury mechanisms, dysregulated myokine secretion reversely aggravates immune-mediated myocyte apoptosis and protein catabolism through multiple intestinal mucosal injury pathways, ultimately forming a bidirectional vicious cycle of intestinal damage and skeletal muscle atrophy (78). Based on this myokine-mediated intestine-muscle interactive injury loop, four typical intestinal pathological alterations are systematically summarized, including intestinal epithelial apoptosis and erosion caused by aberrant CD8^+^ T cell infiltration, persistent inflammatory storm and disrupted intestinal epithelial barrier induced by excessive pro-inflammatory cytokine release, microcirculatory ischemia and lactic acid accumulation resulting from sustained microvascular inflammatory damage, as well as extracellular matrix deposition and rectal-specific fibrotic lesions driven by excessive activation of immunosuppressive cells (79). Each type of pathological injury corresponds to distinct and quantifiable imaging features, which enable intuitive acquisition of tissue-level pathological details via diverse imaging modalities. Based on the complete myokine-regulated molecular injury network, precise correlations can be established between multi-modal quantitative imaging parameters and molecular pathological changes. This strategy achieves a full translational chain from internal injury mechanisms to non-invasive clinical imaging evaluation, thereby enabling accurate severity assessment and dynamic therapeutic monitoring of myokine-related intestinal injury (80).

## 4 Application of imaging technology in the evaluation and monitoring of colorectal injury

### 4.1 Colonoscopy combined with narrow-band imaging technology

Previous studies have verified that myokine dysregulation mediates intestinal inflammation, vascular injury, and fibrosis, and these pathological alterations can be quantitatively identified via multimodal imaging. Accordingly, this chapter matches each myokine-regulated injury mechanism with corresponding imaging biomarkers, thereby enabling imaging-based evaluation of the severity of myokine-associated intestinal injury and dynamic monitoring of the efficacy of muscle-protective interventions (81).Colonoscopy combined with narrow-band imaging (NBI) is the core imaging modality for evaluating immune-mediated injury to the colorectal mucosal layer. Colonoscopy, the gold standard for direct visualization of the colorectal mucosa, allows for intuitive and real-time inspection of the intestinal lumen. NBI enhances the visualization of blood vessels and mucosal patterns through spectral filtering, enabling a high-resolution display of early epithelial damage and microvascular changes. The combination of these two techniques facilitates a high-resolution assessment of early epithelial injury (82).

**Figure 3:**
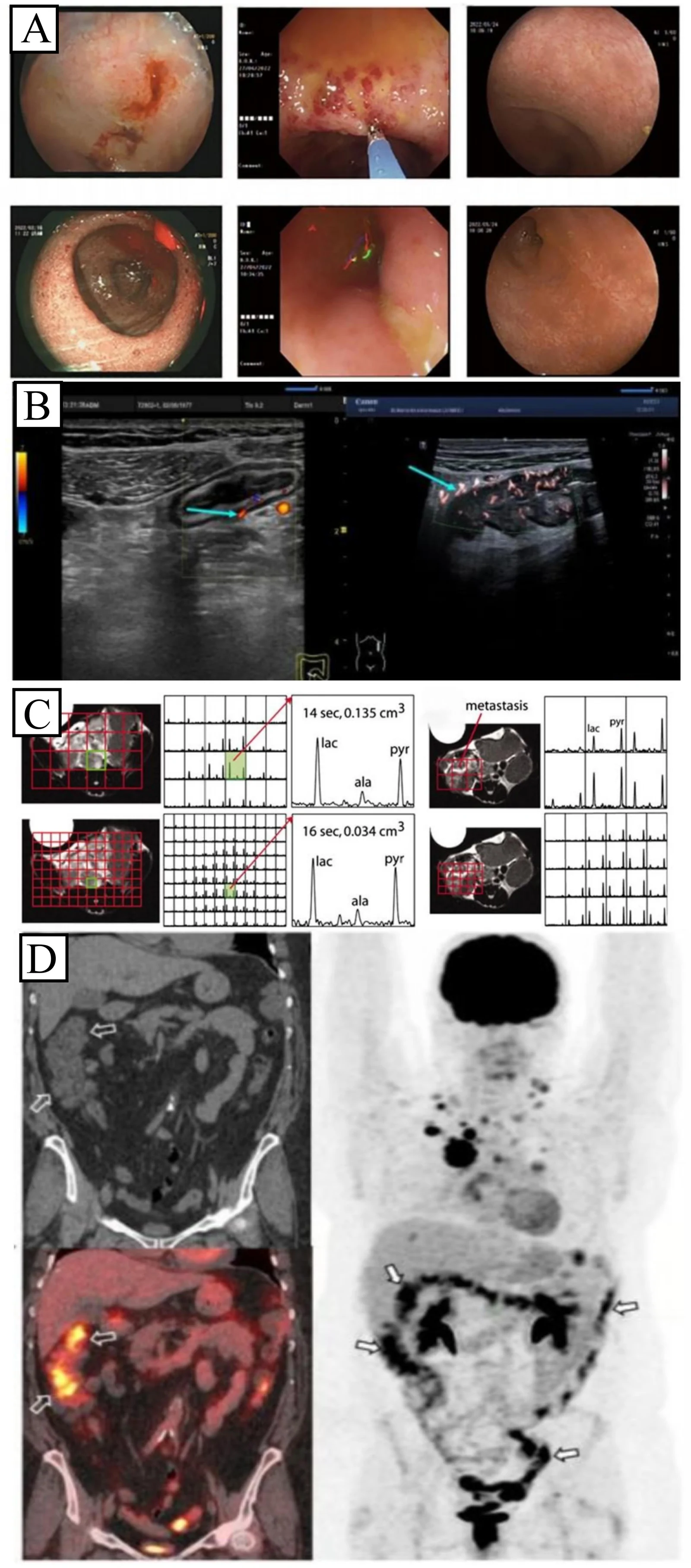
Application of multiple imaging techniques for myokine-associated immune-related colorectal injury. **(A)** Serial NBI colonoscopy presents mucosal hyperemia, erosion and ulceration in PD-1 inhibitor-induced colitis combined with Cytomegalovirus infection (83). **(B)** Color doppler EUS shows thickened intestinal wall and hypervascular signals indicating transmural inflammation and early fibrosis (84). **(C)** Accelerated MRSI visualizes pyruvate, alanine and lactate metabolic distribution. **(D)** FDG positron emission tomography-computed tomography fusion locates hypermetabolic segments with massive immune cell infiltration (85).

CD8^+^ T cells activated by ICIs frequently induce apoptosis of intestinal epithelial cells and focal erosions. Serial NBI colonoscopy of toripalimab-treated patients with concurrent Cytomegalovirus infection displays mucosal hyperemia, punctate erosion and progressive superficial ulceration linked to CD8^+^ T cell cytotoxic injury (**Figure 3A**). Furthermore, under the influence of inflammatory cytokine storms and immune cell infiltration, the crypt architecture is often disrupted and may form crypt abscesses; the magnified imaging advantage of NBI allows for the direct visualization of crypt morphological distortion or loss (**Table 2**) (95).

**Table 2:** Imaging evaluation methods for colorectal injury.

Imaging technique	Detection target	Detection metrics	Corresponding mechanism	Clinical value	Limitations	References
Colonoscopy + NBI	Mucosal vascular injury, erosion, and crypt damage	Mucosal hyperemia score; Erosion area; Crypt integrity	CD8^+^ T cell-mediated epithelial cytotoxicity; inflammatory burst and crypt destruction	Early detection of immune mucosal injury; treatment tolerance prediction	Unable to detect deep lesions; results operator-dependent	(86, 87)
EUS + SWE	Intestinal deep ECM fibrotic lesions	Submucosal thickness (mm); Tissue elastic modulus (kPa)	CAF activation; excessive collagen deposition; reduced tissue compliance	Distinguish inflammatory edema from irreversible intestinal fibrosis	Requires specialized equipment; steep operational learning curve	(88–90)
Contrast-enhanced CT/MRI	Vascular injury, mucosal hypoperfusion and metabolic disorders	Bowel wall enhancement (HU/SI); Edema score (0–3); Vascular stenosis rate (%)	Endothelial injury; immune microthrombosis; mucosal ischemia and hypoxia	Monitor ischemic injury; differentiate inflammation; guide vascular protection therapy	Radiation and contrast agent risks; limited subtle metabolic detection ability	(91, 92)
PET-CT (^18^F-FDG)	Immune infiltration and intestinal glycolytic activity	SUVmax; SUVmean; Metabolic tumor volume	CD8^+^ T cell aggregation; enhanced glycolysis; lactic acid accumulation	Quantify inflammatory scope; distinguish immune injury from infection	Radiation exposure; limited diagnostic specificity	
Radiomics + AI Model	Multi-modal feature mining and ICI-CRI risk prediction	First/second-order texture features; Wavelet/Gabor features; AUC value	Inflammatory heterogeneity; ischemic necrosis; fibrotic microstructure remodeling	Stratify disease risk; predict immune toxicity; guide individualized therapy	Dependent on large datasets; lack of unified standards and external validation	(93, 94)

In addition to lesion identification, colonoscopy combined with NBI enables inflammation assessment and prognosis prediction using quantitative indicators. The mucosal hyperemia score reflects the degree of vascular dilation mediated by tumor necrosis factor-alpha (tumor necrosis factor-α(TNF-α) and IL-6). The quantitative erosion area indicates the extent of CD8^+^ T cell-mediated cytotoxicity, and crypt integrity reflects the status of mucosal repair in the intestine (96). These parameters can be correlated with clinical biomarkers (such as pro-inflammatory cytokine levels and the degree of immune cell infiltration) to comprehensively evaluate the severity and recovery potential of immune-related mucosal inflammatory lesions, thereby providing a quantitative basis for clinical grading and intervention. When imaging reveals diffuse inflammatory changes and crypt destruction rather than focal infiltration, the findings are more suggestive of immune-related injury than of tumor recurrence (86). Quantitative endoscopic indicators, including mucosal congestion score, erosion area, and crypt integrity, can indirectly reflect the degree of systemic myokine dysregulation and serve as non-invasive imaging biomarkers for myokine-associated ICI-CRI.

### 4.2 Endoscopic ultrasound and shear wave elastography technology

Endoscopic Ultrasound (EUS) is a key imaging modality for evaluating deep tissue damage in the colorectum associated with immunotherapy. This technology utilizes high-frequency ultrasound integrated with an endoscopic probe, enabling real-time visualization of the five-layer structure of the intestinal wall and accurate identification of alterations, such as wall thickening, layer blurring, and echo enhancement in the mucosa, submucosa, and muscularis propria. This provides a direct basis for differentiating between inflammation and fibrosis (97). As shown in **Figure 3B**, color Doppler EUS images show diffuse intestinal wall thickening, blurred five-layer wall structure and marked intramural hyper vascular signals consistent with Limberg grade 3–4 inflammatory hyperemia. This intuitively presents the deep structural changes in the intestinal wall in immunotherapy-related colorectal injury, reflecting the impact of pathological conditions, such as abnormal Extracellular Matrix (ECM) deposition, on the intestinal wall structure. Under the influence of ICIs, patients may experience abnormal ECM remodeling and myofibroblast activation, leading to continuous deposition of type I collagen, increased tissue stiffness, and reduced intestinal wall compliance, thereby inhibiting mucosal repair (98). EUS can detect intestinal wall thickening and enhanced blood flow signals, which reflect local inflammatory responses and vascular proliferation. The integration of Shear Wave Elastography (SWE) has established an Endoscopic Ultrasound-Shear Wave Elastography (EUS-SWE) platform, allowing for the quantitative evaluation of ECM deposition through the measurement of the elastic modulus (kPa) of the intestinal wall, which objectively reflects tissue stiffness and the progression of fibrosis (99).

In clinical practice, the submucosal thickness and elasticity values obtained via EUS-SWE are key parameters: a thickness > 4 mm combined with an elasticity > 25–30 kPa indicates significant ECM deposition and structural fibrosis driven by activated CAFs; a moderate thickness with an elasticity of 10–20 kPa mostly corresponds to the inflammatory edema stage, which is potentially reversible (100). This technology can determine whether a lesion has progressed to an established fibrotic stage by dynamically monitoring elasticity trends, thereby providing a basis for monitoring the efficacy of antifibrotic therapies. Concurrently, EUS-SWE complements colonoscopy with NBI; the former focuses on evaluating deep mechanical structures, while the latter reflects superficial mucosal damage (101). These imaging and quantitative parameters closely correspond to the mechanisms of abnormal ECM deposition, myofibroblast activation, and fibrosis formation, as summarized in **Table 2**, providing a comprehensive foundation for mechanistic research and clinical assessment of colorectal immune-related injury (**Table 2**). Based on the established EUS-SWE quantitative detection system, quantitative parameters such as intestinal wall thickness and elastic modulus can indirectly reflect the systemic level of myokine dysregulation and serve as non-invasive imaging biomarkers for deep fibrotic injury in myokine-associated ICI-CRI.

### 4.3 Enhanced CT/MRI technology

Enhanced Computed Tomography (CT) and Magnetic Resonance Imaging (MRI) are the primary non-invasive imaging modalities for evaluating colorectal vascular permeability, perfusion status, and metabolic changes following immunotherapy. Through intravenous injection of iodine-based or gadolinium-based contrast agents, dynamic observation of intestinal wall enhancement patterns, vascular distribution, and edema is enabled. When combined with multiphase perfusion analysis, a comprehensive assessment of blood flow and metabolism can be achieved (102).

After ICI treatment, the overactivation of CD8^+^ T-cells and neutrophils can cause vascular endothelial injury and immune-mediated microthrombosis, leading to microvascular stenosis and local ischemia. CT and MRI can sensitively detect reduced intestinal wall perfusion, delayed enhancement, and edema in the intestinal tract. Furthermore, Magnetic Resonance Spectroscopy, as part of an MRI examination, can detect lactate accumulation, reflecting tissue hypoxia and metabolic dysfunction (103). Based on animal experiment results utilizing accelerated Magnetic Resonance Spectroscopic Imaging, the color overlay in **Figure 3C** intuitively displays the spatial distribution of metabolites across different regions, and the spectral comparison further reveals metabolic disparities between tumor and non-tumor areas (104). This imaging strategy demonstrates high spatial resolution and tissue characterization capability, indicating that such technologies hold significant reference value for the early assessment of metabolic disorders in immune-related colorectal injury, particularly showing potential advantages in detecting lactate accumulation, hypoperfusion, and alterations in tissue boundaries.

In clinical applications, key quantitative parameters include the degree of intestinal wall enhancement (Hounsfield Unit/Signal Intensity), edema score (grade 0-3), and mesenteric vascular stenosis rate (%). Reduced enhancement or delayed perfusion suggests impaired blood flow, a stenosis rate >50% indicates severe vascular injury, and the edema score reflects the degree of inflammatory activity (105). Enhanced CT and MRI can enable the early identification of immune-related ischemic injury and distinguish it from simple inflammatory reactions, thereby guiding decisions on whether to initiate anticoagulant or vascular protective therapy (**Table 2**). Based on the multi-dimensional quantitative parameters derived from contrast-enhanced CT/MRI, quantitative indicators including intestinal wall enhancement degree, edema score, mesenteric vascular stenosis rate, and lactate metabolism-related magnetic resonance spectroscopy parameters can indirectly reflect the extent of *in vivo* myokine dysregulation, and can serve as non-invasive imaging biomarkers for evaluating myokine-mediated vascular and metabolic disorder-dominant ICI-CRI.

### 4.4 PET-CT imaging for evaluating immunotherapy-related immune cell infiltration and metabolic activity injury

Positron Emission Tomography-Computed Tomography (PET-CT) is an important functional imaging modality for evaluating immune-related colorectal injury by assessing immune cell infiltration and metabolic activity (22). This technology integrates the metabolic imaging capability of PET with anatomical localization of CT. Using ^18^F-fluorodeoxyglucose (^18^F-FDG) as a glucose metabolism tracer, the metabolic activity and intensity of the immune response in colorectal tissue can be quantitatively reflected through the standardized uptake value (SUV) (32).

Following ICI treatment, activated CD8^+^ T cells extensively infiltrate the lamina propria of the intestinal mucosa. To sustain their cytotoxic functions, these cells significantly upregulate glycolysis, leading to increased glucose consumption. This heightened metabolic activity results in elevated local ^18^F-FDG uptake and a marked increase in SUV (106). Concurrently, the enhanced energy metabolism of other inflammatory cells, such as macrophages and neutrophils, further amplifies these signals (**Table 2**). Therefore, PET-CT can intuitively identify metabolic “hotspots” corresponding to sites of immune cell aggregation, thereby delineating the extent and activity of immune-mediated damage in the heart.

As shown in **Figure 3D**, integrated Maximum Intensity Projection, coronal CT and PET-CT fusion images display segmental colorectal hypermetabolic signals labeled by white arrows, representing massive infiltration of glycolysis-active CD8^+^ T cells and macrophages This fusion intuitively visualizes regions with heightened immune cell infiltration and metabolic activity, providing further confirmation of ICI-CRI. PET-CT metabolic quantitative parameters, such as SUV, can indirectly reflect the status of systemic myokine secretion disorder and serve as non-invasive imaging biomarkers for myokine-regulated immune cell infiltration-dominant ICI-CRI.

### 4.5 Multidimensional evaluation technology based on radiomics and artificial intelligence

Radiomics combined with artificial intelligence (AI) provides a novel approach for the precise assessment of immunotherapy-related colorectal injuries. Its core principle involves extracting high-dimensional quantitative features from multimodal images, such as enhanced CT and MRI, and utilizing algorithmic modeling to achieve disease diagnosis, prognosis prediction, and treatment response evaluation, thereby overcoming the limitations of traditional subjective visual assessment (37).

This process primarily consists of three stages: the first is data preprocessing and segmentation. After collecting multimodal images, procedures such as registration, standardization, and denoising are performed to mitigate the variance arising from different imaging equipment and contrast protocols. Subsequently, the region of interest is accurately delineated, either manually or via AI-based automatic segmentation algorithms (requiring a Dice coefficient ≥ 0.85), to ensure precise correspondence between the target area and the reference tissue (107). The second stage is feature extraction and feature selection. The extracted features encompass first-order statistical features (e.g., mean CT value, standard deviation), second-order texture features (e.g., Gray-Level Co-occurrence Matrix contrast, correlation), and higher-order transformed features (e.g., from wavelet or Gabor filtering). These features reflect subtle structural alterations associated with inflammatory heterogeneity, ischemic necrosis, and ECM fibrosis (108). The third stage involved model construction and validation. Predictive models are built using algorithms such as Least Absolute Shrinkage and Selection Operator, Random Forest, Support Vector Machines, or deep learning architectures (e.g., ResNet). Their performance was then evaluated through multicenter cross-validation with metrics including Area Under the Curve, sensitivity, and specificity to ensure model robustness and generalizability (38).

In clinical practice, radiomics models can facilitate risk stratification for immune-related toxicity, predict treatment response, and support individualized clinical decision-making (109). The feature dimensions and model performance metrics correspond to the mechanisms summarized in table, providing a quantitative foundation for the intelligent analysis of multimodal images in immune-related colorectal injury (**Table 2**). High-dimensional radiomic texture features and predictive efficacy metrics of AI models can indirectly characterize the degree of systemic myokine dysregulation, serving as non-invasive comprehensive monitoring biomarkers for intelligent screening of the occurrence risk and severity evaluation of myokine-associated ICI-CRI.

## 5 Repair strategies for immunotherapy-induced colorectal injury

Existing intestinal repair strategies can reversely regulate myokine secretion in skeletal muscle and block the vicious cycle of muscle wasting and intestinal injury. From multiple dimensions including inflammation, gut microbiota, vascular metabolism, fibrosis, and skeletal muscle, this chapter elaborates stratified intervention strategies that achieve dual regulation of intestinal repair and myokine homeostasis. Intestinal damage induced by ICIs primarily results from the excessive activation of T helper 1 cell/T helper 17 cell and other immune cells, leading to disruption of the intestinal epithelial barrier and localized inflammatory responses. This triggers an extensive immune reaction. Repair mechanisms play a crucial role in the self-healing process of the intestine during ICI-CRI. By understanding and intervening in these repair mechanisms, it is possible to effectively mitigate damage, promote mucosal healing, and prevent fibrosis in the stomach.

### 5.1 Identification and rapid intervention of initial-stage inflammation

Multimodal techniques, including endoscopy, functional imaging, and radiomics-based artificial intelligence (AI), enable quantitative evaluation of myokine imbalance-induced intestinal inflammatory activity, microcirculatory impairment, and fibrotic staging, thereby achieving precise stratification of injury risk (110). Based on such imaging stratification results, the following section systematically elaborates stage-specific stratified repair and intervention strategies. While restoring intestinal mucosa and blocking inflammatory cascades, all therapeutic regimens can reversely modulate the secretion of skeletal muscle-derived interleukin-6 (IL-6), irisin, and IL-15, remodel myokine homeostasis, and interrupt the vicious cycle linking muscle wasting and intestinal injury. Collectively, these approaches construct an integrated diagnostic and therapeutic framework consisting of pathological mechanism clarification, noninvasive imaging stratification, and individualized comprehensive repair. To further clarify the theoretical basis of stratified intervention strategies, the core pathogenesis and repair mechanisms of early immune-mediated colitis (IMDC) are summarized in the following section. The core goal of early intervention for IMDC is to achieve precise blockade before inflammatory amplification occurs. The initiating mechanism primarily stems from an imbalance between excessive immune activation and disruption of the epithelial barrier. T helper 1 cell/T helper 17 cells release a large number of pro-inflammatory factors, such as IL-6 and tumor necrosis factor-α (TNF-α), leading to the degradation of tight junction proteins, occludin and Zonula Occludens-1. This results in luminal antigen leakage, further activating local immune responses and forming a positive inflammatory feedback loop. Maintaining the stability of the intestinal stem cell microenvironment is critical during this process (43). Su Bing’s team demonstrated that MRISC upregulates R-spondin1 through the ROS–MAP3K2–ERK5–KLF2 signaling axis and activates the Wingless/Integrated pathway to maintain the regenerative potential of Leucine-Rich Repeat-Containing G-Protein-Coupled Receptor 5 stem cells (111). Therefore, early IMDC treatment should aim to control inflammation while protecting the metabolic interactions between epithelial stem cells and stromal cells to prevent the progression of inflammation to fibrosis. Based on the above principles of early intervention for intestinal anti-inflammation and stem cell protection, the stratified combined metabolic intervention regimen established in this study effectively upregulates the expression of protective myokines, including IL-15 and irisin, while inhibiting the release of muscle-derived pro-inflammatory IL-6. This strategy reverses myokine dysregulation and blocks the intestinal-muscle interactive inflammatory vicious cycle, thereby alleviating early ICI-associated colorectal injury while preserving systemic anti-tumor immunity and achieving dual therapeutic benefits.

### 5.2 Second-line and subsequent immunosuppressive therapy

For patients with glucocorticoid-refractory or glucocorticoid-dependent IMDC, the therapeutic objective is to achieve sustained control of inflammation while maximizing the preservation of antitumor immune function, thereby creating conditions for the safe reintroduction of subsequent immunotherapy. The key lies in selecting targeted immunosuppressive pathways based on the patient’s specific inflammatory molecular phenotype and immune microenvironment characteristics of the patient. Pathologically, glucocorticoid resistance is often associated with persistent activation of pro-inflammatory signals, such as IL-6, TNF-α, and Full name: Interleukin-17A, as well as aberrant homing of effector T-cells. Concurrently, increased expression of Mucosal Addressin Cell Adhesion Molecule-1 on the mesenteric vascular endothelium promotes alpha4 beta7 integrin integrin-mediated lymphocyte infiltration (112).

Stratified targeted biologics constitute the backbone of second-line and salvage regimens for IMDC (**Table 3**), encompassing TNF-α neutralizers, gut-tropic integrin antagonists, IL-12/23 inhibitors, and next-generation Janus Kinase (JAK) inhibitors for multi-refractory patients. Close surveillance for latent opportunistic infections and anti-pneumocystis preventive therapy are mandatory during JAK inhibitor administration.

**Table 3:** Treatment strategies and clinical trials for ICI-associated colorectal immune injury.

Repair phase	Therapeutic mechanism	Biomarkers	Research methods	Drug	Target type	Clinical trial ID	Trial year	Phase	Participant count	Outcomes	Refs.
Inflammation Initiation Phase	Non-specific anti-inflammatory suppression	CRP, ESR, Fecal Calprotectin	Serology & Fecal Tests & Endoscopy	Corticosteroids	Glucocorticoid Pathway	NCT04305145	2020	II	42	Expected 68% clinical remission rate at 2 weeks in corticosteroid group	(87, 88)
	Localized inhibition of gut inflammation via suppression of lymphocyte migration	α4β7 Integrin expression, Lymphocyte subsets, Fecal Calprotectin	Flow Cytometry & Fecal Tests & Endoscopic Biopsy	Vedolizumab	α4β7 Integrin, Gut-specific	NCT04797325	2021	II	82	Expected 42% reduction in lamina propria T-cell infiltration on biopsy; Reduced systemic immunosuppression-related adverse events (6%)	(89, 90)
Secondary Immunosuppressive Therapy	Blocking TNF-α pro-inflammatory cascade, Inhibiting T-cell migration	TNF-α levels, IL-6, T-cell subsets, Endoscopic mucosal healing	Serology & Flow Cytometry & Endoscopy	Infliximab/Vedolizumab	TNF-α/α4β7 Integrin	NCT04407247	2020	I/II	100	Effectively alleviate, ICI-colitis symptoms; Significant post-treatment decrease in TNF-α, IL-6 levels	(91, 92)
	Early addition of TNF inhibitor to shorten inflammation duration, reduce steroid dependence	TNF-α levels, CRP, Time to clinical remission	Serology & Clinical Scores	Infliximab + Steroids vs. Steroids alone	TNF-α Inhibition	NCT05947669	2023	III	195	Expected >30% reduction in steroid dependence rate within 6 months	(91, 93)
Gut Microecological Reconstruction	Reconstruction of donor microbiota, inhibition of pro-inflammatory bacteria, improvement of colitis and ICI efficacy	Gut Microbiota, 16S rRNA, Metabolomics	Fecal Sequencing & Multi-omics	Fecal Microbiota Transplantation (FMT)	Gut Microbiota, Immunomodulation	NCT03772899	2019	I	20	30% of patients restored response to ICI; 55% colitis symptom remission rate; No severe adverse events	(94, 113)
	Application for ICI-colitis treatment	Gut microbiota structure, IL-10/Treg levels	Fecal Sequencing & IHC & Cytokine Detection	Fecal Microbiota Transplantation (FMT)	Gut Microbiota Reconstruction	NCT04729322	2021	II	15	60% patient colitis symptom remission; Increased IL-10 levels and Treg cell proportion	(114, 115)
	Standardized FMT products or synthetic microbial consortia combined with ICI	Microbiome signature, Serum metabolites	Fecal Sequencing & Metabolomics & Clinical Scores	LND101 (Standardized FMT)	Gut Microbiota, Immunomodulation	NCT06623461	2025	II	128	Expected to increase tumor response rate while reducing colitis incidence	(116, 117)
Vascular and Metabolic Regulation	Improving intestinal mucosal oxygen supply	HIF-1α, Lactate, Intestinal blood flow	Tissue Biopsy & Metabolic Assays & Imaging	HBOT, Glutamine, Enteral Nutrition	Vascular Microenvironment, Metabolic Support	NCT05987852	2024	II	126	2-fold increase in mucosal HIF-1α expression; 35% reduction in lactate levels; Improved intestinal blood flow; 40% mucosal healing rate	(118, 119)
Fibrosis Inhibition and Structural Reconstruction	Inhibiting fibroblast activation	TGF-β signaling, Collagen deposition, Bowel wall stiffness	MRI & Histology & Biomarkers	Anti-TGF-β/Anti-fibrotic agents	TGF-β/Smad, TWIST1	NCT04467580	2020	I/II	59	20% reduction in bowel wall stiffness (MRI); Suppressed TGF-β pathway activity; Reduced collagen deposition	(120, 121)

Uniform quantitative criteria have been established to guide safe ICI reinitiation, which include grade 1 or milder gastrointestinal adverse events, low-dose glucocorticoid maintenance, and endoscopic remission defined by a Mayo score ≤1. Serial measurements of fecal calprotectin and autoantibody profiling are recommended for longitudinal relapse risk surveillance (122). These multi-line targeted immunosuppressive regimens efficiently interrupt IL-6-triggered myogenic inflammatory cascades, upregulate the production of protective myokines (irisin and IL-15), and disrupt the vicious sarcopenia-intestinal injury cycle, thereby laying a favorable foundation for the subsequent safe reinitiation of immune checkpoint inhibitor therapy (123).

### 5.3 Reconstruction of intestinal microecology

Intestinal dysbiosis is an important driving factor for the persistence and recurrence of immune checkpoint inhibitor-related colitis (124). IMDC patients often exhibit a decreased Firmicutes-to-Bacteroidetes ratio and abnormal proliferation of Proteobacteria, leading to reduced synthesis of short-chain fatty acids, impaired regulatory immunity, and exacerbated inflammatory responses. Intestinal flora imbalance is closely related to the continuous elevation of IL-6 and IL-8 and is a key link in the chronicization of the disease and progression to fibrosis (125).

The core of intestinal microecology reconstruction lies in the bidirectional regulation of the flora-immune axis: Lactobacillus and Bifidobacterium can produce butyrate through the fermentation of dietary fiber, inhibit Histone Deacetylase activity, increase the percentage of Treg cells, and enhance the expression of occludin and Zonula Occludens-1, thereby repairing the mucosal barrier. Propionate reduces the secretion of IL-6 by macrophages through the G-Protein Coupled Receptor 43 signal, thereby inhibiting the spread of inflammation (126). Akkermansia muciniphila enhances the antigen presentation of dendritic cells and the antitumor effect of ICIs, maintaining intestinal balance (127). Based on the abovementioned mucosal protective mechanisms regulated by gut microbiota and mediated by short-chain fatty acids (SCFAs), gut microecological reconstruction regimens can restore SCFA homeostasis. On the one hand, such regimens suppress the secretion of muscle-derived pro-inflammatory cytokine IL-6; on the other hand, they upregulate the expression of skeletal muscle protective myokines, including IL-15 and irisin, thereby breaking the vicious cycle linking gut dysbiosis, myokine imbalance and intestinal mucosal injury.

### 5.4 Comprehensive regulation of vascular and metabolic homeostasis

**Figure 4:**
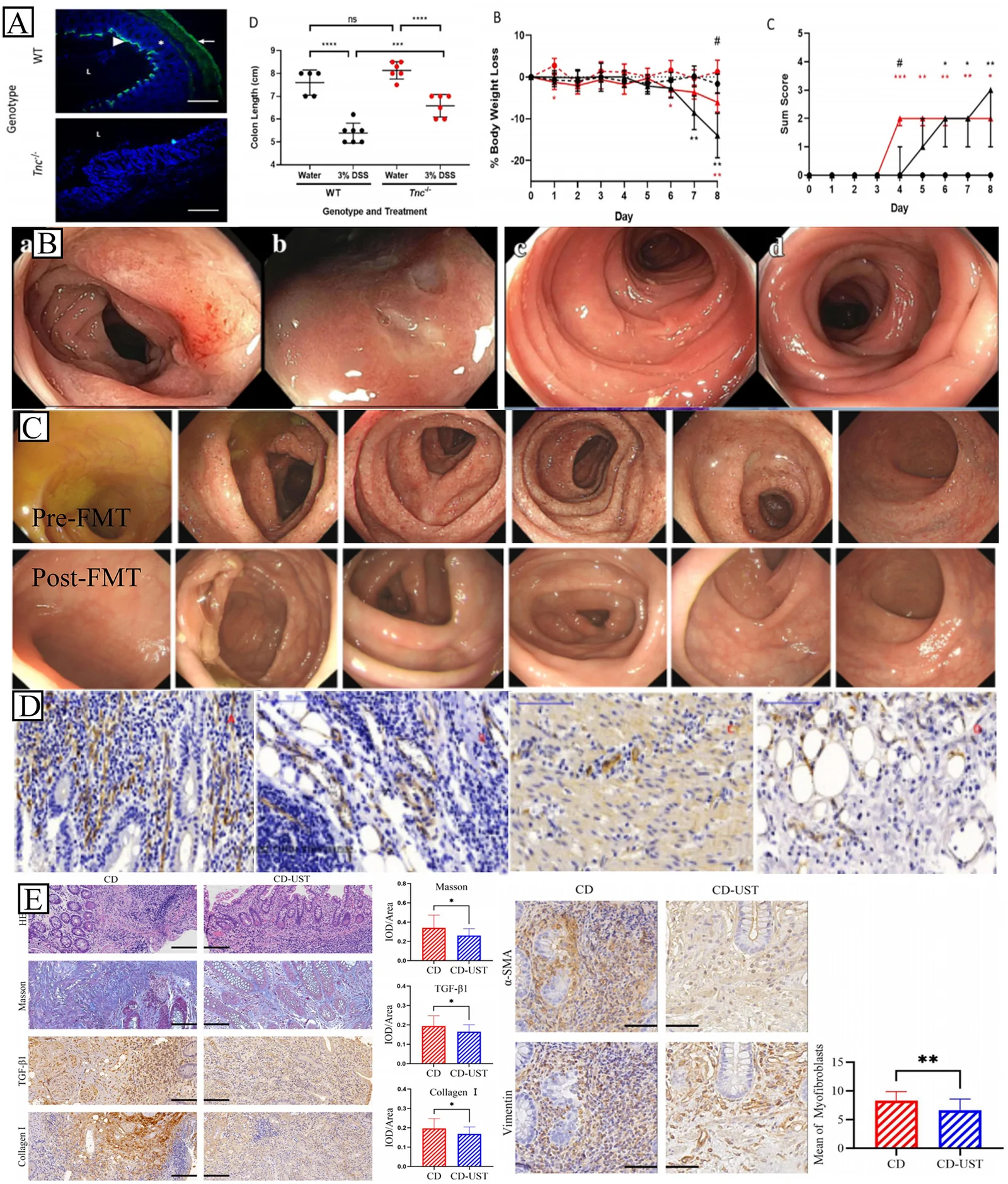
Multimodal pathological and omics evidence of intestinal fibrosis and inflammatory injury in ICI-associated colitis and Crohn’s disease. **(A)** Serial NBI colonoscopy images of toripalimab-induced immune colitis combined with Cytomegalovirus infection, showing progressive mucosal erosion, extensive ulceration and complete mucosal repair after standardized intervention (131). **(B)** Colonoscopic comparison before and after second-line treatment targeting the Interleukin-12/Interleukin-23 (IL-12/23) pathway in glucocorticoid-refractory cases (93). **(C)** Immunohistochemical staining of full-thickness intestinal specimens for Vascular Endothelial Growth Factor Receptor 2, revealing enhanced angiogenesis across mucosa, submucosa, muscularis propria and subserosa in active inflammatory lesions (115). **(D)** Single-cell transcriptome Uniform Manifold Approximation and Projection clustering and differential gene volcano plots of intestinal myofibroblasts stratified by Cysteine Rich Protein 1 expression, illustrating profibrotic transcriptional signatures in high- KDEL Cysteine Rich Protein 1 MF (132). **(E)** Immunofluorescence double staining of tenascin-C and epithelial marker Epithelial Cell Adhesion Molecule in DSS-induced murine colitis, with prominent tenascin-C deposition localized to ulcerative mucosal injury sites (133). *p < 0.05, **p < 0.01, ***p < 0.001, and ****p < 0.0001; ns indicates no statistically significant difference. Asterisks indicate comparisons between the DSS-treated groups and their corresponding genotype-matched water control groups. # indicates p < 0.05 for DSS-treated Tnc^-^/^-^ mice versus DSS-treated WT mice. The white asterisk in the immunofluorescence image indicates the muscularis mucosa and does not represent statistical significance.

**Figure 5:**
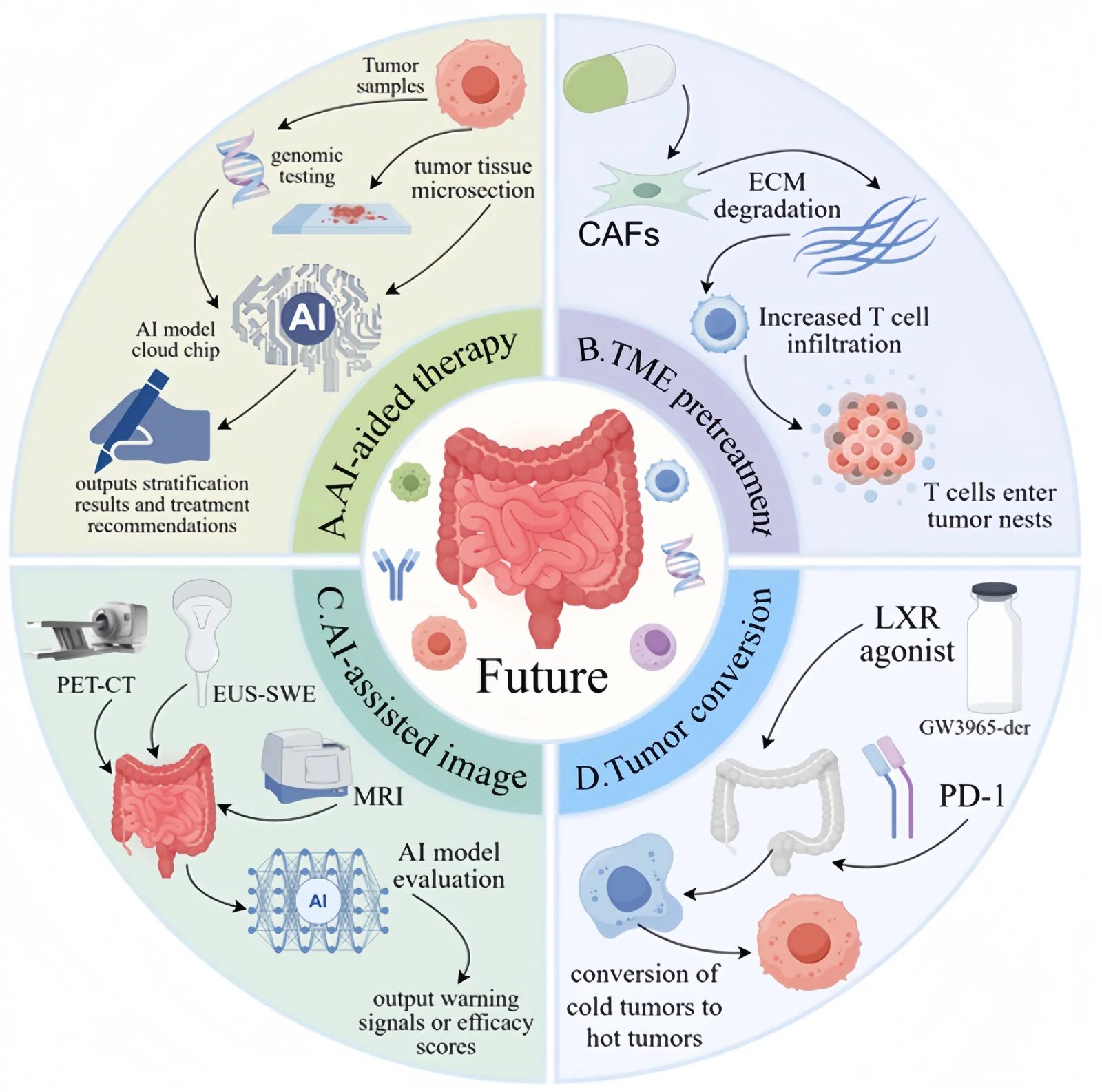
Future outlook of rectal cancer treatment. **(A)** Artificial intelligence models integrate multi-omics data to generate patient stratification results and personalized treatment recommendations; **(B)** Pre-treatment modulation of the TME promotes ECM degradation and enhances T cell infiltration; **(C)** AI-assisted imaging evaluation outputs early warning signals or efficacy scores; **(D)** Combination therapy achieves the conversion from “cold tumor” to “hot tumor” phenotypes.

In the intermediate and advanced stages of IMDC, a vicious cycle often develops, involving microvascular perfusion disorders, metabolic imbalances, and impaired tissue repair processes. Long-term inflammatory damage leads to compromised vascular endothelial function, local hypoxia, and inadequate nutrient supply, forming the core pathological basis for failed structural recovery (128). Therefore, therefore, the therapeutic objective should shift from inflammation control to a dual approach of improving perfusion and optimizing metabolism, thereby restoring local microcirculation and energy homeostasis. At the molecular level, sustained high expression of TNF-α and IL-1β activates endothelial cells, increases vascular permeability, and induces platelet adhesion, leading to microthrombosis and reduced perfusion, which can decrease intestinal mucosal blood flow by approximately 30% (129). Persistent hypoxia further inhibits mitochondrial oxidative phosphorylation and energy substrate utilization, whereas chronic inflammation disrupts glutamine and butyrate metabolism, impairing stem cell regeneration and epithelial repair capacity. Systematic multi-modal intervention strategies are developed to interrupt the above pathological cascade, covering microcirculation rescue, metabolic substrate supplementation, anti-fibrotic pharmacotherapy and structural endoscopic intervention. Targeted anticoagulant and enteral nutritional interventions can reverse mucosal hypoperfusion and relieve intestinal metabolic overload, while glutamine and butyrate synergistically stabilize epithelial barriers via the G-Protein Coupled Receptor 109A–IL-18 axis (130). Colonoscopic images before and after IL-12/23-targeted second-line therapy visually demonstrate mucosal healing outcomes in glucocorticoid-refractory ICI-colitis patients (**Figure 4B**). Preclinical evidence from Dextran Sulfate Sodium (DSS)-induced colitis models (**Figure 5D**) confirms that microvascular-targeted natural compounds such as sweroside effectively reverse endothelial injury and normalize tissue energy metabolism, providing translational evidence for vascular-metabolic therapeutic regimens. A series of anti-fibrotic agents including harmine and long-term vedolizumab maintenance can restrain excessive extracellular matrix deposition, and auxiliary supplementation of vitamin A and zinc further facilitates epithelial regeneration and matrix remodeling (134). Full-thickness intestinal immunohistochemical staining for Vascular Endothelial Growth Factor Receptor 2 distinctly captures pervasive angiogenesis across mucosal, submucosal, muscular and subserosa layers during active inflammatory episodes (**Figure 4C**). For patients progressing to irreversible intestinal stenosis, hierarchical structural interventions ranging from endoscopic balloon dilation to segmental bowel resection are implemented according to intestinal wall stiffness quantified by Endoscopic Ultrasound-Shear Wave Elastography (EUS-SWE) (**Figure 5E**). Single-cell transcriptome analysis with UMAP clustering and differential volcano plots identifies unique profibrotic transcriptional signatures in KDELC1-high intestinal myofibroblasts (**Figure 4D**). Combined EUS-SWE and Narrow Band Imaging (NBI) endoscopy enable precise differentiation between reversible inflammatory edema and permanent fibrotic lesions, and serum Extracellular Matrix (ECM)-related biomarkers can be applied to dynamically adjust anti-fibrotic treatment schemes. Serial NBI colonoscopy images record the whole disease trajectory of toripalimab-induced immune colitis combined with Cytomegalovirus infection, covering progressive mucosal erosion, extensive ulceration and final complete mucosal repair after standardized intervention (**Figure 4A**). EUS-derived elastic modulus and Positron Emission Tomography-Computed Tomography (PET-CT) standardized uptake value (SUV) serve as non-invasive quantitative imaging biomarkers to dynamically monitor the amelioration of microcirculatory dysfunction and regression of intestinal fibrosis, facilitating longitudinal therapeutic efficacy evaluation for combined vascular and metabolic regulatory regimens. Double immunofluorescence staining of tenascin-C and epithelial marker Epithelial Cell Adhesion Molecule in DSS-induced colitic mice verifies massive tenascin aggregation restricted to ulcerative mucosal injury sites, which acts as a specific biomarker for fibrotic lesion formation (**Figure 4E**). Collectively, this integrated therapeutic paradigm combining microcirculation restoration, metabolic supplementation and anti-fibrotic intervention alleviates hypoxia-triggered skeletal muscle catabolism, suppresses the secretion of pro-inflammatory myokine IL-6, and boosts the biosynthesis of protective myokines (IL-15 and irisin), thereby disrupting the vicious feedback loop linking vascular-metabolic disturbance, dysregulated myokine signaling and progressive rectal fibrosis (135).

### 5.5 Role and regulation of skeletal muscle in immune-mediated colitis

In the pathological process of IMDC, skeletal muscle serves not only as an important tissue for movement and metabolism but also plays a crucial role in immune responses (136). Research indicates that skeletal muscle can regulate the activity of immune cells by releasing muscle-derived cytokines (myokines), such as IL-6, IL-15, and muscle growth factors, thereby influencing systemic inflammatory states (137). This function is particularly important in patients with IMDC, as disease progression is often accompanied by systemic inflammation and muscle wasting (i.e., cachexia). Therefore, understanding the interplay between the skeletal muscle and immune system is essential for developing more comprehensive treatment strategies (138). The Fluorine-18 fluorodeoxyglucose (^18^F-FDG) SUV measured via PET-CT within skeletal muscle compartments enables direct quantification of whole-body muscular metabolic activity, which indirectly mirrors systemic dysregulated secretion of myokines including IL-6, irisin and IL-15. ^18^F-FDG PET/CT can quantitatively assess glucose metabolic activity in skeletal muscle via SUVmax values, and distinguish physiological and pathological muscle metabolic abnormalities (139). Skeletal muscle orchestrates a central regulatory cascade termed the “skeletal muscle–myokine–intestinal injury” axis. Myokines are categorized into two functional subgroups: protective mediators (IL-15 and irisin) and the pathogenic pro-inflammatory factor IL-6. Each subtype modulates intestinal immune populations, epithelial barrier integrity and stromal remodeling via distinct independent signaling pathways, acting as pivotal upstream regulators governing the initiation and progression of ICI-CRI (59). Exercise and nutritional interventions centered on this muscle-intestinal regulatory axis are capable of remodeling systemic myokine profiles: they upregulate protective IL-15 and irisin while blunting excessive IL-6 release, simultaneously mitigating ICI-induced intestinal lesions and sarcopenia, and ultimately achieving dual clinical benefits consisting of preserved anti-tumor immunity and alleviated gastrointestinal immune toxicities (140).

#### 5.5.1 Protective myokines: IL-15 and irisin alleviate ICI-CRI injury

IL-15 and irisin are two representative protective myokines secreted by intact skeletal muscle, which relieve ICI-associated intestinal mucosal damage through independent signaling axes. Muscle-derived IL-15 activates the JAK/Signal Transducer and Activator of Transcription 5 (STAT5) pathway in circulating CD8^+^ T cells and NK cells, enhances their antitumor cytotoxicity while limiting their aberrant cross-reactivity against intestinal self-antigens; meanwhile, IL-15 downregulates intestinal Treg infiltration and reduces the secretion of immunosuppressive Transforming Growth Factor-β (TGF-β), balancing mucosal immune response intensity. IL-15 exerts dual immunomodulatory effects via the JAK/STAT5 signaling cascade: it potentiates the tumoricidal capacity of CD8^+^ T and NK cells while restraining intestinal Treg infiltration and TGF-β secretion, thereby preventing aberrant immune cross-reactivity against intestinal epithelial cells (141). Irisin blocks the CSF-1/STAT3 cascade in lamina propria macrophages to suppress M2-type Tumor-Associated Macrophage (TAM) polarization, lowers local Interleukin-10 (IL-10) accumulation, and inhibits persistent nuclear factor κB (NF-κB) inflammatory activation in intestinal epithelial cells (142). Additionally, irisin upregulates tight junction proteins occludin and claudin-1 to restore impaired intestinal barrier function, indirectly reducing Lipopolysaccharide translocation and secondary inflammatory amplification after ICI treatment. As for irisin, it interrupts inflammatory cascades through two complementary mechanisms: it suppresses the polarization of M2-type tumor-associated macrophages to relieve local immunosuppressive microenvironment and concurrently blocks epithelial NF-κB signaling to upregulate tight junction proteins and restore intestinal barrier integrity. Both myokines can reverse the vicious cycle of sarcopenia-intestinal inflammation in rectal cancer patients receiving immunotherapy (64).

#### 5.5.2 Pathogenic myokine IL-6 drives the aggravation of ICI-CRI

Unlike IL-15 and irisin, sustained high levels of muscle-released IL-6 act as a pathogenic mediator that exacerbates ICI-CRI, relying on dual JAK/STAT3 and NF-κB signaling pathways. Under sarcopenic conditions, atrophic myofibers overproduce IL-6, which enters the systemic circulation and accumulates in intestinal mucosal tissue. Excess IL-6 continuously activates epithelial NF-κB signaling, promoting the release of pro-inflammatory TNF-α and IL-1β to trigger inflammatory cytokine storms (143). Meanwhile, IL-6-mediated JAK/STAT3 activation accelerates intestinal fibroblast transformation into myofibroblasts, boosting collagen deposition and submucosal fibrosis. Under sarcopenic conditions, overproduced IL-6 simultaneously activates two pathogenic signaling axes: it triggers an epithelial inflammatory storm through NF-κB cascades, while driving myofibroblast activation via JAK/STAT3 signaling, ultimately facilitating intestinal collagen deposition and progressive fibrotic lesions (18). Notably, IL-6 exhibits concentration-dependent bidirectional effects: physiological low concentrations maintain intestinal immune tolerance, whereas high levels induced by muscle loss disrupt mucosal homeostasis and amplify ICI-triggered autoimmune intestinal damage (17).

#### 5.5.3 Exercise and nutritional intervention modulate myokine secretion to reverse ICI-CRI

Targeted exercise and nutritional support can remodel the secretion profile of muscle-derived myokines, simultaneously boosting protective IL-15/irisin expression and restraining excessive IL-6 production, which represents a non-drug auxiliary strategy for ICI-CRI management. Moderate resistance training stimulates myofiber synthesis and upregulates irisin and IL-15 transcription via the Peroxisome Proliferator-Activated Receptor Gamma Coactivator 1α (PGC-1α) pathway, while reducing resting muscle IL-6 release. Moderate resistance training elevates the transcription of protective myokines by activating the skeletal muscle PGC-1α signaling pathway, thereby restraining the over-secretion of pathogenic IL-6 from myofibers at the source (144). Nutritional supplementation with high-quality protein, vitamin D and branched-chain amino acids alleviates muscle protein degradation, prevents sarcopenia, and stabilizes the balanced secretion of myokines (145). Combined exercise-nutrition regimens can not only mitigate intestinal inflammatory erosion and fibrosis, but also preserve the antitumor immune efficacy of ICIs, avoiding the clinical dilemma of long-term hormone therapy suppressing systemic antitumor immunity (146).

#### 5.5.4 Circulating myokines as predictive and monitoring biomarkers for ICI-CRI

Peripheral blood levels of IL-15, irisin and IL-6 can serve as non-invasive circulating biomarkers for predicting the occurrence, severity and recovery trend of ICI-CRI before and during immunotherapy. Low baseline irisin and IL-15 combined with elevated IL-6 levels are strongly correlated with higher risks of grade 2–4 IMDC. Peripheral myokine panels prior to immunotherapy enable high-risk patient screening; low baseline levels of IL-15 and irisin accompanied by elevated IL-6 confer substantially increased risks of severe intestinal injury (147). Dynamic monitoring of myokine concentrations can assist multi-modal imaging (PET-CT, EUS-SWE) to stratify intestinal injury severity: elevated serum IL-6 corresponds to severe mucosal inflammation, while reduced irisin predicts progressive intestinal fibrosis (148). After intervention with exercise, nutrition or immunosuppressive agents, the recovery of myokine balance can reflect mucosal repair efficacy and guide the safe timing of ICI reintroduction, providing a novel quantitative indicator for individualized clinical monitoring. Collectively, skeletal muscle-derived myokines form a core regulatory hub linking sarcopenia, systemic inflammation and ICI-induced colorectal injury. Targeted modulation of myokine homeostasis through muscle protection interventions provides an innovative multi-target therapeutic direction for balancing antitumor immunity and reducing immune-related gastrointestinal toxicity in rectal cancer patients (149).

### 5.6 Unmet clinical demands and novel myokine-based interventional strategies

Current ICI-associated colorectal injury treatment guidelines are derived from general inflammatory bowel disease standards, with unmet demands in individualized risk stratification, targeted therapy and efficacy-toxicity balance; the myokine-mediated muscle-intestinal axis provides novel theoretical targets to solve these clinical bottlenecks.

Traditional clinical evaluations only rely on intestinal local indicators (endoscopic morphology, inflammatory biochemical markers) to judge mucosal recovery and ICI reintroduction timing, ignoring systemic metabolic and immune signatures. Circulating IL-6, irisin and IL-15 reflect host inflammatory repair status, and their expression profiles can distinguish superficial epithelial healing from genuine mucosal remission, supporting multimodal imaging-combined individualized ICI resumption assessment to balance antitumor efficacy and gastrointestinal toxicity. However, major limitations persist: no unified myokine diagnostic cutoffs for populations stratified by age, muscle volume and Mismatch Repair status; single myokine indexes fail precise risk stratification; multi-marker models combining myokines with NBI, EUS-SWE and PET-CT only pass single-center retrospective validation and lack universal standardized monitoring protocols (150).

Current therapy mainly relies on stepwise single-agent immunosuppression. Glucocorticoids or single biologics merely relieve local intestinal inflammation and cannot break the sarcopenia-myokine disorder-intestinal injury vicious cycle. Multi-combination schemes (biologics + flora reconstruction + metabolic nutrition) can jointly normalize myokine secretion and block systemic inflammation, reducing monotherapy’s immunosuppressive side effects and extending remission duration. Yet standardized exercise-nutrition matching protocols are unavailable, and large multicenter prospective data to define safe exercise intensity and nutritional dosage for patients with sarcopenia, acute ulcer or progressive rectal fibrosis remain absent. Besides, myokine-targeted drug translation lags: routine drugs only indirectly adjust myokine levels, while IL-6 neutralizers and long-acting IL-15/irisin agonists are not clinically accessible, restricting simultaneous mucosal protection and antitumor immunity maintenance (151).

No dedicated regimens exist for ICI-induced rectal fibrosis, and routine anti-inflammatory drugs cannot reverse abnormal collagen deposition and intestinal wall sclerosis. Pirfenidone inhibits fibroblast and TGF-β pathways to slow fibrosis, but its clinical value, optimal administration window and safety in rectal ICI-fibrosis lack systematic verification. Myokines exert dual control over fibrosis: sustained high IL-6 amplifies fibrotic cascades, while irisin suppresses stromal overdeposition. Combined myokine regulators and anti-fibrotics may reverse early fibrosis and delay irreversible structural damage, though relevant evidence mostly comes from cell and animal studies, with insufficient human tissue data quantifying correlations between serum myokines, intestinal elasticity and fibrosis staging (152).

Steroid resistance is a primary cause of chronic refractory ICI-colitis, yet reliable early predictive biomarkers are lacking. Combined detection of myokines and TNF-α gene polymorphisms can identify steroid-resistant high-risk patients before treatment, enabling timely switch to second-line biologics and avoiding persistent inflammation and tissue injury from ineffective steroid therapy.

Crucially, traditional broad immunosuppressants carry an inherent tradeoff: they ease intestinal autoimmunity but weaken systemic antitumor immunity and worsen tumor control. In contrast, skeletal muscle homeostasis interventions exert bidirectional myokine regulation: they suppress muscle-derived pro-inflammatory signals to alleviate gut injury while enhancing antitumor immunity, simultaneously lowering gastrointestinal toxicity and preserving/improving ICI efficacy, forming a novel individualized full-cycle risk-benefit management framework.

In summary, myokines link sarcopenia, systemic inflammation, rectal immune injury and progressive fibrosis. Myokine-centered biomarker screening, combined medication and muscle-protective adjuvant interventions can fill multiple gaps in current diagnosis and treatment, serving as a frontier research direction for precise, dual-benefit individualized therapy against immunotherapy-related rectal injury (146).

### 5.7 Cutting-edge research directions based on myokines

This review has systematically elaborated the molecular mechanisms underlying the skeletal muscle-intestinal injury regulatory axis governed by three core myokines (IL-15, irisin and IL-6), and validated that multiple interventions including exercise rehabilitation, nutritional supplementation, targeted immunotherapeutics and intestinal microecological reconstruction can alleviate ICI-associated colorectal injury via restoring homeostatic myokine secretion. In view of the clinical translational bottlenecks summarized above, this subsection outlines prospective translational and basic research directions centered on the myokine regulatory network, providing systematic research frameworks for subsequent mechanistic exploration and clinical practice.

Benefiting from rapid advances in multi-omics, radiomics and artificial intelligence, future investigations can integrate skeletal muscle imaging indicators, tumor molecular subtypes, circulating myokines and multi-omic signatures to construct intelligent, individualized risk prediction models, and formulate standardized cut-off values of myokines stratified by distinct patient populations (153). Such systems enable precise pre-immunotherapy screening and early warning of patients at high risk of ICI-CRI (154). In terms of myokine-targeted drug translation, research priorities include developing intestinal sustained-release formulations and long-acting myokine mimetics, such as IL-6 neutralizing agents and stable agonists of IL-15 and irisin (155). Non-pharmacological combinatorial regimens combining structured exercise training and mild myokine-modulating interventions will also be explored to perfect the multi-modal framework for restoring balanced myokine profiles (156).

To further dissect the bidirectional muscle-intestinal crosstalk, follow-up work will unravel reciprocal regulatory loops between myokines and gut microbiota, and clarify key signaling cascades through which short-chain fatty acids modulate myokine synthesis and secretion in skeletal muscle (157). These findings will consolidate the theoretical basis for combined interventions consisting of fecal microbiota transplantation, prebiotic supplementation and muscle-protective regimens (158). Longitudinal prospective cohorts will be established to dynamically track fluctuating myokine levels across various therapeutic stages, and formulate standardized criteria for ICI reinitiation centered on recovered myokine homeostasis to reduce disease relapse risk. Combined with precise imaging modalities such as EUS elastography tailored to the unique fibrotic anatomical features of the rectum, clinical translational trials will be conducted to systematically verify whether myokine-targeted therapy can restrain aberrant intestinal collagen deposition and reverse or delay irreversible fibrotic progression (159). Stratified research will also be performed on heterogeneous subgroups including elderly patients, cancer cachexia sufferers and subjects with severe postoperative muscle wasting, to develop graded exercise rehabilitation and personalized nutritional schemes, and expand the clinical applicability of integrated myokine regulatory strategies (140).

In conclusion, myokines act as the central regulatory hub linking sarcopenia, systemic chronic inflammation and the onset and progression of ICI-CRI, possessing remarkable value for basic research and clinical translation. In-depth mechanistic dissection and multi-center prospective cohort validation targeting current clinical obstacles, along with translational research following the above proposed research avenues, will facilitate the establishment of standardized comprehensive management protocols that balance anti-tumor immune responses and prevention of colorectal immune toxicities, and propel precision immunotherapy for rectal cancer into an innovative paradigm featuring coordinated skeletal muscle-intestinal regulation.

## 6 Conclusion and challenge

In the context of rectal cancer immunotherapy, this study systematically reviewed the composition of the TME and its dynamic remodeling mechanisms, and further explored the characteristics of colorectal mucosal and wall injuries induced by immune interventions. Immunotherapy targeting immune checkpoints has dramatically transformed the clinical management of rectal cancer, yet ICI-CRI remains a major clinical obstacle limiting therapeutic benefits. Research indicates that while immune activation is central to antitumor therapy, its “double-edged sword” effect cannot be overlooked. At the pathological level, immune cell subsets and Extracellular Matrix (ECM) deposition within the TME collectively influence the intensity and direction of the immune response. At the metabolic level, the Warburg effect, local hypoxia, and lactate accumulation contribute to immune suppression and mucosal damage. At the vascular level, microvascular endothelial dysfunction and inflammatory factor cascades form a vicious cycle that impedes tissue repair (71). Distinct from previous reviews that merely concentrate on intestinal lesions and TME, the present work innovatively establishes an integrated skeletal muscle–myokine–intestinal injury regulatory axis (160). This review systematically delineates multiple molecular pathways whereby three pivotal myokines (IL-6, IL-15) and irisin exert bidirectional modulation over anti-tumor immunity and ICI-induced gastrointestinal toxicity, and identifies myokines as critical mediators bridging sarcopenia, systemic inflammation and intestinal mucosal damage, which constitutes the core novelty that differentiates this manuscript from analogous published literature (48). Skeletal muscle plays a pivotal role in this process. Evidence indicates that skeletal muscle can modulate immune cell activity and shape the systemic inflammatory status through the secretion of myokines. Accordingly, muscle wasting is not only associated with the magnitude and efficacy of immune responses but may also exacerbate immune-related tissue injury, thereby reinforcing this vicious cycle. Moreover, preserving skeletal muscle health and function is critical for optimizing the overall benefits of immunotherapy. Appropriate exercise interventions and nutritional support can improve muscle mass and quality, which may enhance patients’ tolerance and responsiveness to immunotherapy. Multimodal imaging and radiomics technologies provide a means to visualize and quantify the spatial heterogeneity of these mechanisms, offering new avenues for early identification, efficacy assessment, and individualized intervention (107). Based on this, scientists have proposed shifting the repair of immunotherapy-related colorectal injury from broad-spectrum immunosuppression to organ-specific immune regulation. Through a multidimensional intervention system—identifying early inflammatory signals, reconstructing intestinal microecology, regulating vascular metabolism, and inhibiting fibrosis—the aim is to optimize treatment tolerance, prolong recurrence-free survival, and enhance the potential for safe reintroduction of immunotherapy (129). Furthermore, with the integrated application of artificial intelligence (AI), multi-omics, and imaging, rectal cancer immunotherapy is advancing into a new era characterized by precision, intelligence, and systematization of treatment.

However, translating these mechanisms and strategies into routine clinical practice faces numerous challenges. First, large-scale prospective cohort data on immune checkpoint inhibitor-related colitis and its subsequent structural damage remain scarce, and many treatment strategies are still in the early phase or retrospective research stages (161). Second, the injury mechanisms are complex and highly heterogeneous, with significant inter-patient variations in immune cell infiltration, microecology, metabolic status, and microvascular permeability, making it difficult to develop universally applicable intervention strategies. Third, although multimodal imaging, radiomics, and c models hold great potential, their standardization, reproducibility, and cost-effectiveness in real-world clinical settings have not been fully validated (108). Additionally, there is no unified high-level evidence to guide immunotherapy reintroduction strategies, including timing, regimen selection, and monitoring recurrence risk. Current clinical surveillance systems lack non-invasive circulating biomarkers capable of early warning against intestinal immune toxicities. Nevertheless, myokines including IL-6, IL-15 and irisin hold great potential as novel stratification biomarkers for ICI-CRI. Unfortunately, standardized detection cutoffs and population-specific judgment criteria have not yet been established, and relevant multi-center validation data remain severely insufficient. Finally, although multidimensional interventions such as microbiota reconstruction, metabolic support, vascular optimization, and fibrosis reversal are theoretically well-founded, their implementation in real-world practice often encounters issues related to patient comorbidities, treatment tolerance, cost, and long-term safety. Current clinical interventions predominantly target intestinal inflammation or tumor immunity in isolation, lacking synergistic therapeutic strategies combining skeletal muscle-targeted modulation with ICI treatment. Muscle-protective regimens such as exercise rehabilitation and nutritional supplementation have not been incorporated into the standardized management workflow of rectal cancer immunotherapy. Furthermore, myokine-targeted agonists and neutralizing agents remain confined to preclinical investigations, resulting in a substantial gap in clinical translation. Looking ahead, the deep integration of AI, multi-omics (genomics, transcriptomics, metabolomics, and microbiomics), and multimodal imaging (Positron Emission Tomography-Computed Tomography (PET-CT), Magnetic Resonance Imaging (MRI), Endoscopic Ultrasound-Shear Wave Elastography (EUS-SWE), and radiomics feature extraction) is poised to become a breakthrough. By developing predictive models based on individual immune and microenvironmental characteristics, effective prediction and management can be achieved (162). Future core research directions will focus on two major translational dimensions of myokine-based applications. First, artificial intelligence-assisted predictive models integrating circulating myokine profiles, multi-omics signatures and multimodal imaging parameters should be developed to establish a pre-treatment risk-stratification system for ICI-induced intestinal toxicity. Defining clinical cut-off values for irisin, IL-15, and IL-6 enables non-invasive, dynamic, and whole-course monitoring of intestinal inflammation and fibrotic progression, and supports biomarker-guided decision-making for ICI reinitiation (153). Second, novel synergistic strategies combining skeletal muscle-targeted modulation with immunotherapy should be explored. Activation of the PGC-1α pathway can upregulate protective myokines, thereby achieving dual therapeutic benefits of muscle protection and enhanced anti-tumor immunity while suppressing IL-6-driven myogenic inflammatory cascades in the intestine (163). As illustrated in **Figure 5**, the future of rectal cancer immunotherapy will towards multidimensional integration and intelligence-driven approaches.

Artificial intelligence (AI) is poised to play a central role in precise diagnosis and personalized treatments. By integrating genomics, transcriptomics, and histopathological data, AI models can achieve accurate patient stratification and generate personalized treatment recommendations, thereby establishing an intelligent closed-loop system for data analysis and therapeutic decision-making. This significantly enhances the efficiency of clinical decision-making and improves the precision of treatment-matching. Myokine-based multi-omic signatures can be incorporated as core predictive variables into artificial intelligence-driven stratification models (153). This strategy compensates for the inherent limitations of conventional tumor markers, which only reflect tumor burden but fail to predict immune-related toxicities, thereby further improving the precision of individualized diagnosis and treatment (164). In terms of immune strategy, plastic remodeling of the TME represents a critical breakthrough (**Figure 5A**) (165). The TME preconditioning strategy illustrated in **Figure 5B** targets CAFs to promote ECM degradation, thus enhancing T cell infiltration and immune response sensitivity. This facilitates the conversion of immunologically “cold” tumors to “hot” tumors, offering a novel therapeutic opportunity for refractory rectal cancer (166). Skeletal muscle preconditioning remodels the tumor immune microenvironment by augmenting IL-15 secretion, thereby reversing the immunosuppressive “cold tumor” phenotype of mismatch repair-proficient/microsatellite stable tumors and providing a novel immune-sensitizing strategy for refractory rectal cancer (167). AI also demonstrates substantial potential in medical imaging. As shown in **Figure 5C**, AI-assisted imaging technology integrates multimodal data from PET-CT, MRI, and EUS-SWE, enabling a real-time quantitative assessment of the tumor metabolic status, blood perfusion, and fibrosis progression. It can generate efficacy scores and early warning signals, providing a scientific basis for monitoring immunotherapy and predicting the treatment outcomes (162). Combining circulating myokine levels with EUS-SWE-derived intestinal wall elasticity and PET-CT metabolic SUV values enables the construction of integrated quantitative myokine-imaging indices, which effectively distinguish reversible inflammatory edema from irreversible rectal fibrosis (168). Furthermore, as depicted in **Figure 5D**, the conversion of cold tumors to hot tumors is a key focus of future research. Through combined immune modulation approaches, including the administration of Liver X Receptor agonists alongside PD-1 inhibitors, it is possible to promote the transition from an immunologically inert to an immunologically active phenotype, enhance antigen presentation and T cell recruitment, and improve the persistence and sensitivity of the immune response (169). Combined muscle-protective interventions and immune transformation regimens can augment ICI-mediated anti-tumor immune responses without exacerbating intestinal immune damage, thereby resolving the clinical trade-off between maximizing immunotherapeutic efficacy and mitigating gastrointestinal toxicity. In summary, the deep integration of AI-driven precise stratification, TME remodeling, intelligent imaging evaluation, and immune conversion strategies will propel rectal cancer immunotherapy into a new era characterized by systemization, intelligence, and accuracy. The present review systematically summarizes the dual regulatory roles of myokines in modulating immunotherapeutic efficacy and immune-associated intestinal toxicity during rectal cancer immunotherapy (170). By proposing the translational potential of myokines as novel biomarkers and the clinical value of muscle-targeted combined immunotherapeutic strategies, this review fills the research gap concerning the neglected skeletal muscle regulatory axis in existing literature, and provides innovative theoretical foundations and prospective directions for subsequent mechanistic investigations, clinical stratified intervention, and novel drug development (171).
